# Comparative proteomics reveal striking differences in biofilm formation and virulence potential of *Gardnerella leopoldii*, *G. piotii*, and *G. vaginalis* isolates

**DOI:** 10.1128/jb.00416-25

**Published:** 2026-07-20

**Authors:** Elinor Shvartsman, Chris Grant, Peter McQueen, Stuart McCorrister, Garrett Westmacott, Paul Sandstrom, Kelly S. MacDonald

**Affiliations:** 1 Department of Medicine, Section of Infectious Diseases, Rady Faculty of Health Sciences, University of Manitoba 8664, Winnipeg, Manitoba, Canada; 2 Department of Medical Microbiology and Infectious Diseases, Rady Faculty of Health Sciences, University of Manitoba 574854, Winnipeg, Manitoba, Canada; 3 JC Wilt Infectious Diseases Research Center, Public Health Agency of Canada 41687, Winnipeg, Manitoba, Canada; National Institutes of Health, Bethesda, Maryland, USA

**Keywords:** *Gardnerella*, bacterial vaginosis, proteomics, biofilms, virulence

## Abstract

Bacterial vaginosis (BV) is a common, recurrent cause of abnormal vaginal discharge that is associated with an increased risk of sexually transmitted infections and adverse pregnancy outcomes. Despite its clinical significance, the pathogenesis of BV remains incompletely understood, limiting the effectiveness of current interventions. Although the *Gardnerella* genus has been proposed to play a pivotal role in BV pathogenesis via biofilm initiation, it was initially considered monotypic. This paradigm shifted with the recognition of species heterogeneity within the *Gardnerella* genus. Here, we examined *in vitro* biofilm growth and proteomes of a representative isolate from each of three *Gardnerella* species: *G. vaginalis*, *G. leopoldii*, and *G. piotii*. *G. leopoldii* and *G. piotii* formed more robust biofilms compared to *G. vaginali*s, with higher biomass, suggesting differences in extracellular matrix composition. Proteomic analysis identified significant differences in functional pathways and virulence proteins among the studied isolates. Cysteine synthase (CysK), implicated in cysteine synthesis and potentially possessing secondary functions, was enriched in the more robust *G. leopoldii* and *G. piotii* biofilms, suggesting a possible role in biofilm formation. Cytolysins were more abundant in the thinner *G. vaginalis* biofilms, whereas immune evasion proteins were more abundant in *G. leopoldii* biofilms. Adhesion and glycogen-degrading proteins varied in type and abundance across species. These findings reveal functional heterogeneity among the tested isolates and provide insight into how certain *Gardnerella* isolates establish biofilms. Targeting virulence factors identified in this study may offer novel strategies to disrupt *Gardnerella* biofilm formation to improve management of BV.

**IMPORTANCE:**

Bacterial vaginosis (BV) is a leading cause of abnormal vaginal discharge, associated with high post-treatment recurrence. *Gardnerella* species play a central role in BV via initiation of polymicrobial biofilms, yet species-specific contributions remain poorly understood. This work demonstrates that tested isolates of *G. leopoldii* and *G. piotii* form more robust biofilms compared to *G. vaginalis* and differentially produce proteins associated with immune evasion, adhesion, and biofilm formation. Certain proteins were enriched in the more robust biofilm producers, suggestive of differences that may contribute to strain-specific biofilm formation. By demonstrating fundamental differences among the studied isolate biofilms, these findings highlight *Gardnerella* heterogeneity and provide a foundation for future studies aimed at targeting *Gardnerella* biofilm formation in BV.

## INTRODUCTION

Bacterial vaginosis (BV) affects 23%-29% of women of reproductive age worldwide and is a leading cause of abnormal vaginal discharge, incurring an annual economic burden exceeding 4 billion USD (1). Despite its high prevalence and the concerning associations with increased risk of sexually transmitted infections (2, 3) and pregnancy complications (4), the pathogenesis of BV remains incompletely understood. Current treatment guidelines include antibiotics with anaerobe coverage (5), which provide short-term relief but have high long-term recurrence rates (6, 7). *Gardnerella* was initially proposed as an etiological agent of BV (8, 9), although it failed to fully fulfill Koch’s postulates given the isolation of *Gardnerella* species from non-BV vaginal samples (10), and failure to consistently reproduce the condition via *in vivo* inoculations (11).

Fluorescent *in situ* hybridization, along with electron microscopy of vaginal biopsies from individuals with BV, has provided compelling evidence for the role of structured *Gardnerella*-dominant polymicrobial biofilms in BV pathogenesis (12). Follow-up investigations revealed that these cohesive *Gardnerella* forms can be transmitted between sexual partners (13). *In vitro* investigations further support these findings, demonstrating a superior biofilm-forming ability of *Gardnerella* species relative to other BV-associated bacteria (14). Several virulence factors are known to exist in *Gardnerella* species, including sialidases, vaginolysin, and adhesins, and these are postulated to play important roles in BV pathogenesis (15). Vaginal epithelial cells studded with adherent bacteria on microscopy (likely representing a biofilm structure) have been linked to BV and given the name “clue cells” for their role in providing a clue to the underlying microbial dysbiosis (16). The presence of clue cells has been included in the Amsel diagnostic criteria for BV, highlighting the importance of this structure to the condition (17). Taken together, these findings point toward a polymicrobial “consortia” etiology of BV, with certain *Gardnerella* species playing a critical role in the establishment of BV, likely via initiation of biofilm formation (18, 19).

Whole-genome sequencing and *cpn*60 microbial profiling have highlighted the genetic heterogeneity within the *Gardnerella* genus (15). This initially led to the recognition of four different *Gardnerella* clades/subgroups (20, 21), and more recently, the delineation of at least thirteen *Gardnerella* genomospecies (22). Given the growing recognition of the heterogeneity within the *Gardnerella* genus, it is pivotal to understand how *Gardnerella* species vary in biofilm formation and virulence properties and thus their potential contributions to BV pathogenesis. The purpose of this study was therefore twofold, with the first objective to examine biofilm formation of representative isolates of *G. leopoldii*, *G. piotii*, and *G. vaginalis,* followed by proteomic analysis of the biofilms to understand the drivers behind the identified differences in biofilm production among these recently recognized distinct *Gardnerella* species.

## RESULTS

### *In vitro* biofilm formation by *G. leopoldii*, *G. piotii*, and *G. vaginalis* isolates

**Fig 1:**
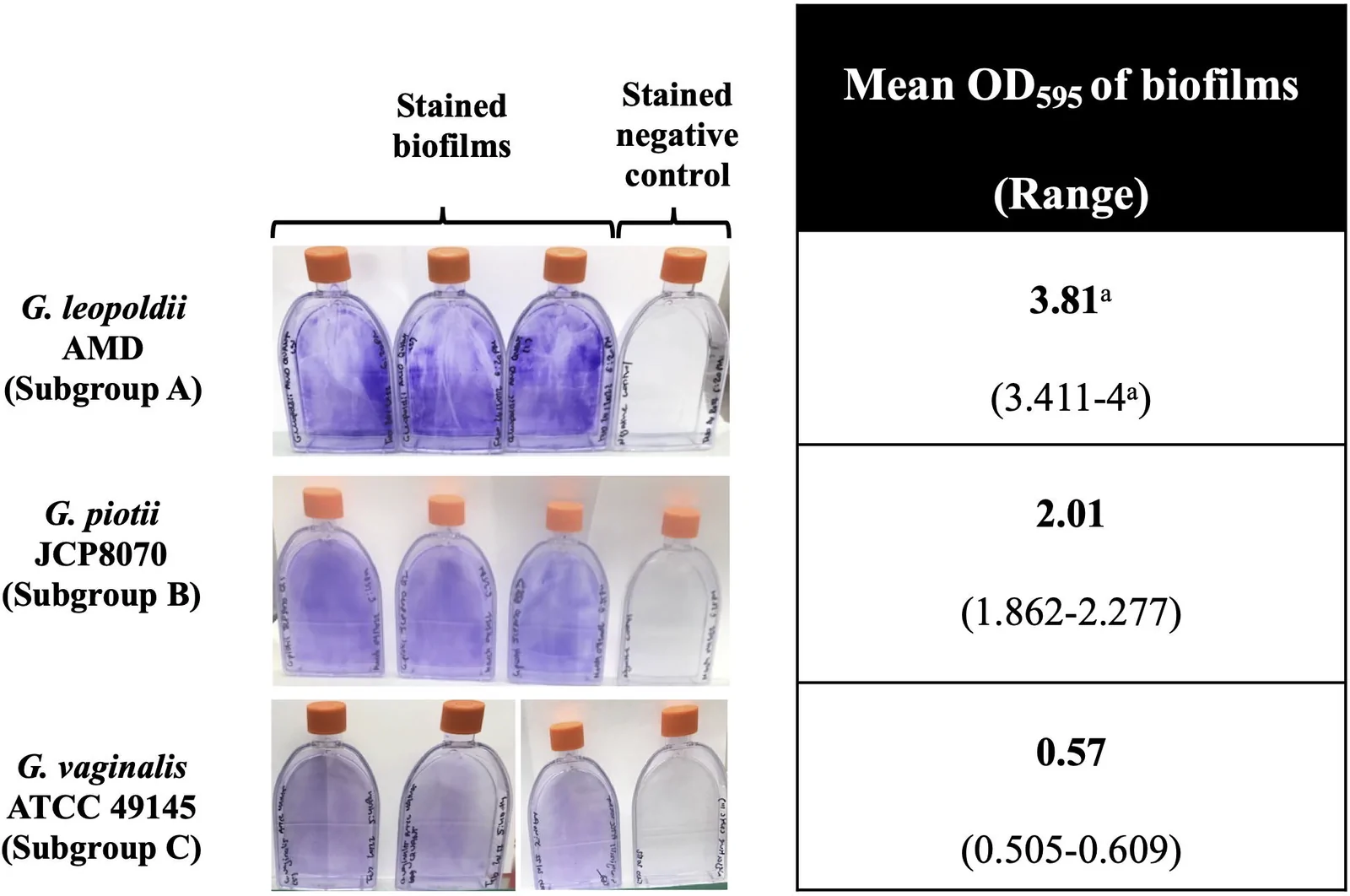
Biofilm quantification using crystal violet staining of *Gardnerella* spp. 48-h biofilms. OD_595_ values were measured in three technical replicates and adjusted by subtracting the OD_595_ values of the negative control flask. ^a^Some biological replicates were out of range (OD_595_ was >4 [upper limit of detection], actual OD_595_ likely higher).

The biomass of 48-hour biofilms was assessed using two methods: crystal violet-based quantification (which does not account for viability) (Fig. 1) and colony-forming unit (CFU) enumeration (which accounts for bacterial viability). Despite having comparable seeding concentrations (Table S1), *G. leopoldii* resulted in the highest biofilm production at 48 h (mean OD_595_ = 3.81, mean CFU = 2.85 × 10^9^), followed by *G. piotii* (mean OD_595_ = 2.01, mean CFU = 1.63 × 10^9^), and with *G. vaginalis* producing the least biofilm (mean OD_595_ = 0.57, mean CFU = 1.33 × 10^8^) (Fig. 1; Table S1).

### Proteomic analysis of *Gardnerella* spp. biofilms

**Fig 2:**
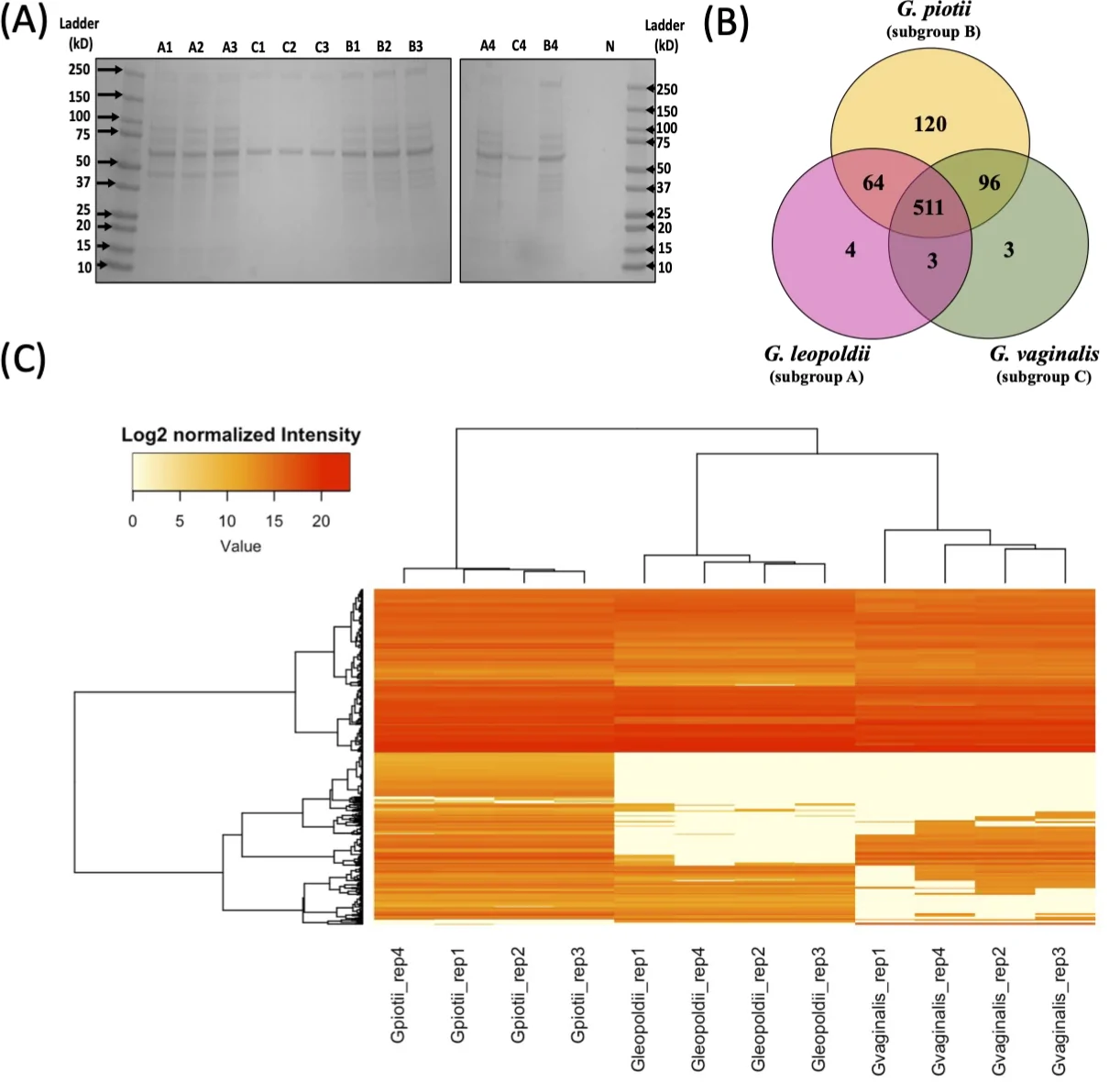
Comparative proteomic analysis of *G. leopoldii* AMD, *G. piotii* JCP8070, and *G. vaginalis* ATCC 49145 biofilms. (**A**) Gradient SDS-PAGE was used to confirm the presence of proteins in the biofilm lysates. Single letters refer to the different *Gardnerella* isolates as follows: A *- G. leopoldii* AMD, B *- G. piotii* JCP8070, C *- G. vaginalis* ATCC 49145. The numbers refer to the corresponding biological replicate. N - negative control (lysis buffer). (**B**) Venn diagram depicting the number of detected unique and shared proteins among *G. vaginalis*, *G. piotii*, and *G. leopoldii* biofilms. The *cpn*60 subgroup classification is noted in brackets. (**C**) Heatmap showing clustering of *Gardnerella* spp. isolate biofilm proteomes by median normalized log2-transformed signal intensity. In cases where no protein was detected, the sample was given the lowest value shown in the heatmap legend (0).

The presence of proteins in *Gardnerella* spp. biofilm lysates was confirmed using sodium dodecyl sulfate-polyacrylamide gel electrophoresis (SDS-PAGE) (Fig. 2A). Of the proteins detected by our analysis, 511 were shared among all three studied isolates, whereas 120 proteins were unique to *G. piotii*, 4 unique proteins were detected in *G. leopoldii*, and 3 unique proteins were detected in *G. vaginalis* (Fig. 2B). In this analysis, a protein was included if it was detected in at least one of the biological replicates for a given *Gardnerella* isolate. Heatmap analysis identified several differentially abundant proteins in the samples, with each of the biological replicates clustering within the specific *Gardnerella* species isolate as expected (Fig. 2C). UniProt accession numbers of detected proteins are supplied in Table S2.

### Differential protein abundance and enrichment analysis of *G. leopoldii* vs *G. vaginalis* biofilms

Overall, 232 proteins were detected at higher abundance, and 194 proteins were detected at lower abundance in *G. leopoldii* compared to *G. vaginalis* (adjusted *P* values ≤ 0.05). The ten significantly most abundant and least abundant proteins are presented in Table 1. Proteins detected at higher abundance included those implicated in cysteine biosynthesis—cysteine synthase (HMPREF3204_00195, CysK) and serine acetyltransferase (HMPREF3204_00196). UDP-glucose 4-epimerase (HMPREF3204_00335), as well as UDP-galactopyranose mutase (HMPREF3204_00366), which are involved in galactose metabolism and galactofuranose synthesis, respectively, were also more abundant in *G. leopoldii* compared to *G. vaginalis*. Tyrosine-tRNA synthetase (HMPREF3204_00035), ATP-dependent Clp protease proteolytic subunit (HMPREF3204_01046), and branched-chain amino acid aminotransferase (HMPREF3204_01110) were also more abundant in *G. leopoldii* compared to *G. vaginalis* (23, 24). The ten least abundant proteins in *G. leopoldii* vs *G. vaginalis* biofilms included an ABC transporter substrate-binding protein (HMPREF3204_00987), alcohol dehydrogenase (HMPREF3204_01235), and pyrroline-5-carboxylate reductase (HMPREF3204_01090). Other proteins that were less abundant in *G. leopoldii* compared to *G. vaginalis* biofilms included DivIVA domain repeat protein (HMPREF3204_00285), which is typically involved with cell division, as well as a single-stranded DNA-binding protein (HMPREF3204_00480), which is involved in DNA replication. An inosine-uridine preferring nucleoside hydrolase (HMPREF3204_00264), hypoxanthine phosphoribosyl transferase (HMPREF3204_00416), and a putative dTDP-4-dehydrorhamnose reductase (HMPREF3204_00354) were also less abundant in *G. leopoldii* biofilms relative to *G. vaginalis* biofilms.

**TABLE 1:** The 10 most significantly differentially abundant proteins in *G. leopoldii* AMD compared to *G. vaginalis* ATCC 49145 biofilms based on log_2_ fold changes (log_2_FC)

Increased abundance			
Gene name	Protein product	log_2_FC	Adj. *P* value
HMPREF3204_00195	Cysteine synthase (EC 2.5.1.47)	7.20	2.13E-07
HMPREF3204_01307	30S ribosomal protein S20	5.11	2.02E-03
HMPREF3204_00196	Serine O-acetyltransferase (EC 2.3.1.30)	4.55	1.33E-09
HMPREF3204_00946	30S ribosomal protein S9	4.06	5.05E-09
HMPREF3204_00335	UDP-glucose 4-epimerase (EC 5.1.3.2)	3.47	4.43E-10
HMPREF3204_00366	UDP-galactopyranose mutase	3.10	6.03E-07
HMPREF3204_00256	L-lactate dehydrogenase (L-LDH) (EC 1.1.1.27)	3.05	9.48E-06
HMPREF3204_00035	Tyrosine-tRNA ligase (EC 6.1.1.1) (Tyrosyl-tRNA synthetase) (TyrRS)	2.92	2.00E-06
HMPREF3204_01046	ATP-dependent Clp protease proteolytic subunit (EC 3.4.21.92) (Endopeptidase Clp)	2.86	5.82E-05
HMPREF3204_01110	Branched-chain amino acid aminotransferase (EC 2.6.1.42)	2.52	3.74E-10

Decreased abundance			
Gene name	Protein product	log_2_FC	Adj. *P* value
HMPREF3204_01083	Lactaldehyde reductase	−9.28	3.33E-10
HMPREF3204_00416	Hypoxanthine phosphoribosyltransferase (EC 2.4.2.8)	−9.27	1.31E-09
HMPREF3204_00354	Putative dTDP-4-dehydrorhamnose reductase	−8.63	1.21E-10
HMPREF3204_00285	DivIVA domain repeat protein	−8.03	2.60E-10
HMPREF3204_01090	Pyrroline-5-carboxylate reductase (P5C reductase) (P5CR) (EC 1.5.1.2) (PCA reductase)	−7.49	3.46E-10
HMPREF3204_00478	30S ribosomal protein S6	−7.46	1.89E-09
HMPREF3204_01235	Alcohol dehydrogenase, propanol-preferring	−7.07	7.48E-11
HMPREF3204_00264	Inosine-uridine preferring nucleoside hydrolase	−6.96	4.09E-08
HMPREF3204_00480	Single-stranded DNA-binding protein (SSB)	−6.95	3.70E-08
HMPREF3204_00987	ABC transporter substrate-binding protein (ABC transporter, substrate-binding protein, family 3)	−6.66	3.74E-10

**Fig 3:**
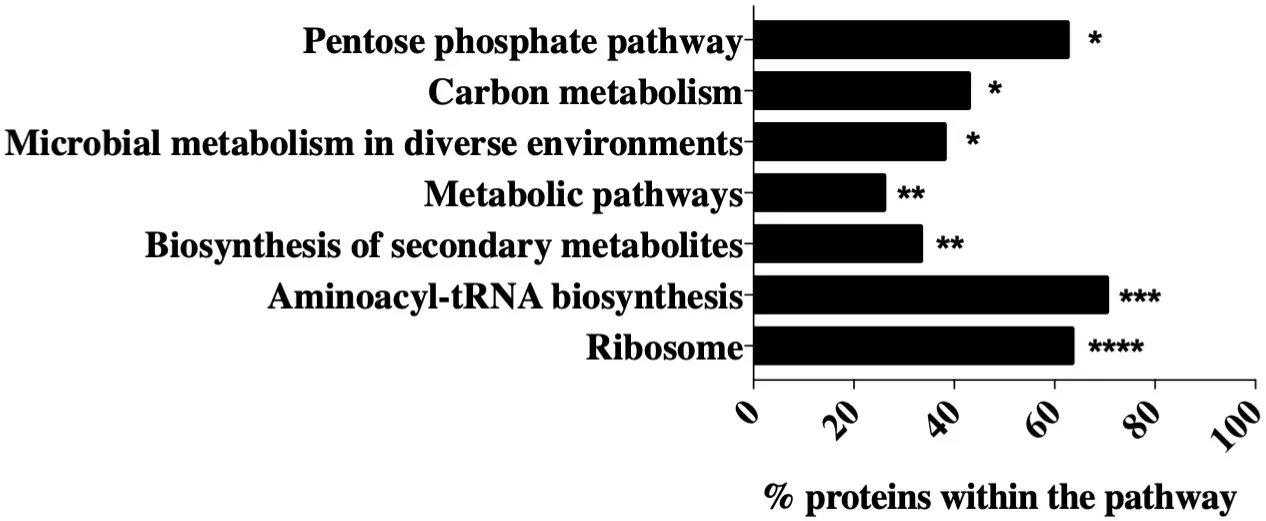
Significantly overrepresented (FDR corrected *P* ≤ 0.05) KEGG pathways in *G. leopoldii* vs *G. vaginalis* biofilms. *P ≤ 0.05, **P ≤ 0.01, ***P ≤ 0.001, ****P ≤ 0.0001.

Enrichment analysis using Kyoto Encyclopedia of Genes and Genomes (KEGG) pathways was performed to understand the functional significance of proteins with higher or lower abundance in *G. leopoldii* vs *G. vaginali*s biofilms. Seven KEGG pathways were overrepresented in *G. leopoldii* vs *G. vaginalis* biofilms using false discovery rate (FDR) corrected *P* ≤ 0.05 (Fig. 3), including pathways involved in carbon metabolism, nucleotide precursor biosynthesis (pentose phosphate pathway), and protein synthesis.

### Differential protein abundance and enrichment analysis of *G. piotii* vs *G. vaginalis* biofilms

Overall, 294 proteins were detected at higher abundance, and 155 proteins were detected at lower abundance in *G. piotii* compared to *G. vaginalis* biofilms (adjusted *P* values ≤ 0.05). The ten significantly most abundant and least abundant proteins in *G. piotii* compared to *G. vaginalis* biofilms are shown in Table 2. Proteins detected at higher abundance included a ribosomal 30S protein (HMPREF3204_01307), cysteine synthase (HMPREF3204_00195), and transporter proteins HMPREF3204_00672 and HMPREF3204_01244. HMPREF3204_01285, which codes for LytR/CpsA/Psr regulator C-terminal domain-containing protein, was also significantly more abundant in *G. piotii* compared to *G. vaginalis* biofilms. HMPREF3204_00792, which codes for a GNAT family acetyltransferase, was also detected at increased abundance in *G. piotii* compared to *G. vaginalis* biofilms. Among the ten least abundant proteins in *G. piotii* vs *G. vaginalis* biofilms were several ABC transporters, including HMPREF3204_00296, HMPREF3204_01209, and HMPREF3204_01301. HMPREF3204_00797, which codes for a putative cholesterol-dependent cytolysin, and [glutamate–ammonia-ligase] adenylyltransferase (HMPREF3204_01135), were also significantly less abundant in *G. piotii* biofilms compared to *G. vaginalis* biofilms.

**TABLE 2:** The 10 most differentially abundant proteins in *G. piotii* JCP8070 compared to *G. vaginalis* ATCC 49145 biofilms based on log_2_ fold changes

Increased abundance			
Gene name	Protein product	log_2_FC	Adj. *P* value
HMPREF3204_01307	30S ribosomal protein S20	6.88	0.000378
HMPREF3204_00195	Cysteine synthase (EC 2.5.1.47)	6.83	7.84E-07
HMPREF3204_01285	LytR/CpsA/Psr regulator C-terminal domain-containing protein	5.31	1.66E-08
HMPREF3204_00420	FemAB family protein	4.81	2.56E-07
HMPREF3204_00792	Acetyltransferase, GNAT family (GNAT family acetyltransferase)	4.65	1.35E-05
HMPREF3204_00826	LPXTG-motif protein cell wall anchor domain protein	4.62	6.85E-05
HMPREF3204_00672	ABC transporter, substrate-binding protein, family 5	4.61	5.79E-07
HMPREF3204_00569	Peptidase	4.49	2.94E-08
HMPREF3204_01244	Transporter, major intrinsic protein family protein	4.38	9.72E-06
HMPREF3204_00615	Glycosyl hydrolase, family 31	4.04	2.97E-09

Decreased abundance			
Gene name	Protein product	log_2_FC	Adj. *P* value
HMPREF3204_00677	Fungal lipase-like domain-containing protein	−10.10	8.06E-09
HMPREF3204_00296	ABC transporter substrate-binding protein (ABC transporter, substrate-binding protein, family 5)	−8.98	2.24E-07
HMPREF3204_00797	Putative perfringolysin O	−8.32	1.12E-09
HMPREF3204_00135	Peptidase	−5.11	6.11E-08
HMPREF3204_01209	ABC transporter glutamine-binding protein GlnH family protein	−3.14	3.79E-10
HMPREF3204_00538	Uncharacterized protein	−3.08	2.31E-07
HMPREF3204_00698	DUF3073 domain-containing protein	−2.94	7.10E-08
HMPREF3204_01301	ABC transporter ATP-binding protein (ABC transporter, ATP-binding protein)	−2.74	1.02E-07
HMPREF3204_01161	Cof-like hydrolase	−2.69	3.57E-06
HMPREF3204_01135	[Glutamate–ammonia-ligase] adenylyltransferase	−2.67	1.89E-05

**Fig 4:**
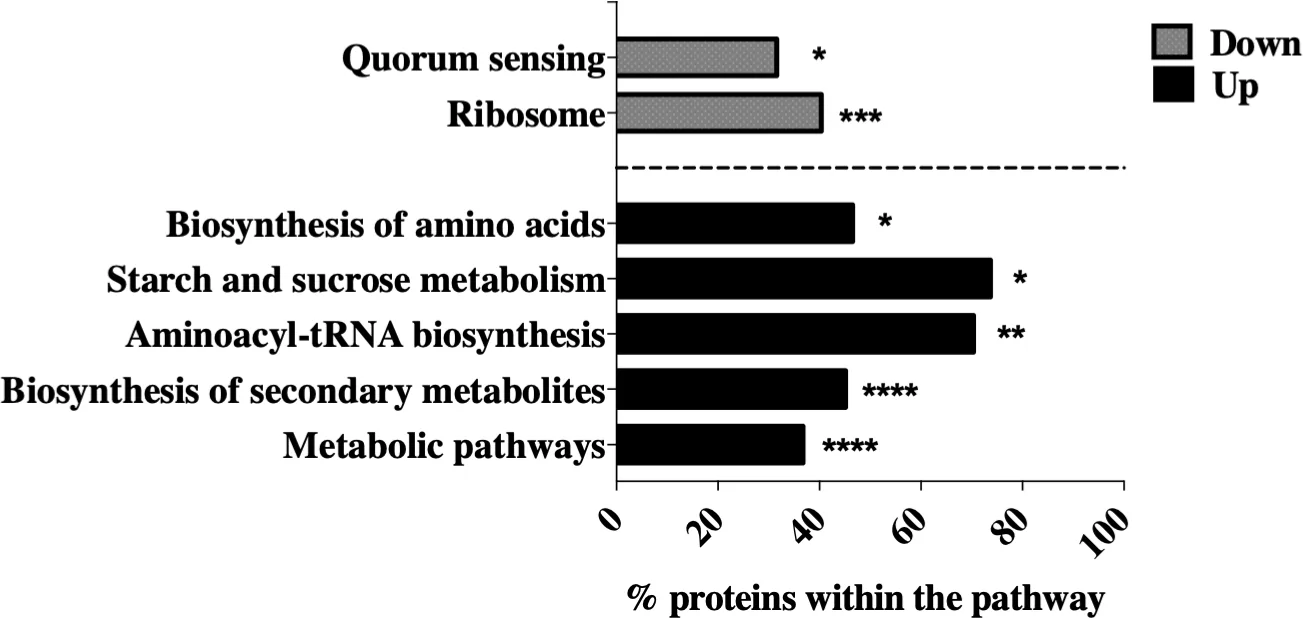
Significantly enriched (FDR corrected *P* ≤ 0.05) KEGG pathways in *G. piotii* vs *G. vaginalis* biofilms. Pathways overrepresented (“Up”) in *G. piotii* vs *G. vaginalis* biofilms are indicated by the black bars, whereas pathways underrepresented (“Down”) in *G. piotii* vs *G. vaginalis* biofilms are indicated by the gray bars. *P ≤ 0.05, **P ≤ 0.01, ***P ≤ 0.001, ****P ≤ 0.0001.

Enrichment analysis using KEGG pathways identified five significantly overrepresented pathways in *G. piotii* vs *G. vaginalis* biofilms (Fig. 4). These included pathways associated with amino acid biosynthesis and sugar metabolism. Two pathways involved in quorum sensing and ribosomal processes were found to be underrepresented in *G. piotii* compared to *G. vaginalis* biofilms (Fig. 4).

### Differential protein abundance and enrichment analysis of *G. leopoldii* vs *G. piotii* biofilms

Overall, 199 proteins were detected at higher abundance, and 257 proteins were detected at lower abundance in *G. leopoldii* vs *G. piotii* biofilms (adjusted *P* values ≤ 0.05). The ten most and least abundant proteins in *G. leopoldii* vs *G. piotii* biofilms are listed in Table 3. HMPREF3204_00037 (argininosuccinate synthase) and HMPREF3204_01135, which codes for glutamate–ammonia-ligase adenylyltransferase, were more abundant in *G. leopoldii* vs *G. piotii* biofilms. Several membrane transporters (HMPREF3204_00113, HMPREF3204_00609) were detected at higher abundance, and others at lower abundance (HMPREF3204_00491, HMPREF3204_00588) in *G. leopoldii* vs *G. piotii* biofilms. A putative cholesterol-dependent cytolysin (HMPREF3204_00797) was also more abundant in *G. leopoldii* vs *G. piotii* biofilms. Least abundant proteins included those associated with nitrogenous base salvaging pathways—inosine-uridine preferring nucleoside hydrolase (HMPREF3204_00264) and hypoxanthine phosphoribosyltransferase (HMPREF3204_00416). The cell division-associated protein HMPREF3204_00285 was also detected at significantly lower abundance in *G. leopoldii* vs *G. piotii* biofilms. Other proteins detected at lower abundance included those coding for metabolic enzymes, such as HMPREF3204_01083, HMPREF3204_01235, and HMPREF3204_01090, as well as the biosynthetic enzyme HMPREF3204_00354.

**TABLE 3:** The 10 most differentially abundant proteins in *G. leopoldii* AMD compared to *G. piotii* JCP8070 biofilms based on log_2_ fold changes

Increased abundance			
Gene name	Protein product	log_2_FC	Adj. *P* value
HMPREF3204_00677	Fungal lipase-like domain-containing protein	8.11	1.08E-08
HMPREF3204_00797	Putative perfringolysin O	8.06	2.56E-10
HMPREF3204_00135	Peptidase	5.99	5.28E-09
HMPREF3204_00113	ABC transporter substrate-binding protein (ABC transporter, substrate-binding protein, family 3)	4.03	6.23E-05
HMPREF3204_01135	[Glutamate–ammonia-ligase] adenylyltransferase	3.29	9.07E-08
HMPREF3204_00366	UDP-galactopyranose mutase	2.96	8.87E-07
HMPREF3204_00609	ABC transporter substrate-binding protein (ABC transporter, solute-binding protein)	2.94	7.63E-08
HMPREF3204_00037	Argininosuccinate synthase (EC 6.3.4.5) (Citrulline–aspartate ligase)	2.91	9.35E-07
HMPREF3204_00105	Nudix hydrolase domain-containing protein	2.75	1.18E-04
HMPREF3204_00197	Acetyltransferase, GNAT family (GNAT family acetyltransferase) (N-acetyltransferase)	2.62	5.22E-08

Decreased abundance			
Gene name	Protein product	log_2_FC	Adj. *P* value
HMPREF3204_00285	DivIVA domain repeat protein	−10.33	5.57E-11
HMPREF3204_00416	Hypoxanthine phosphoribosyltransferase (EC 2.4.2.8)	−10.13	7.93E-10
HMPREF3204_01083	Lactaldehyde reductase	−9.03	3.27E-10
HMPREF3204_00354	Putative dTDP-4-dehydrorhamnose reductase	−8.71	8.30E-11
HMPREF3204_01235	Alcohol dehydrogenase, propanol-preferring	−8.01	2.52E-11
HMPREF3204_01090	Pyrroline-5-carboxylate reductase (P5C reductase) (P5CR) (EC 1.5.1.2) (PCA reductase)	−7.86	2.66E-10
HMPREF3204_00491	Transporter, major facilitator family protein	−7.58	3.91E-08
HMPREF3204_00588	ABC transporter substrate-binding protein (ABC transporter, solute-binding protein)	−7.21	4.36E-07
HMPREF3204_00264	Inosine-uridine preferring nucleoside hydrolase	−7.19	3.29E-08
HMPREF3204_01081	LacI family transcriptional regulator (sugar-binding domain protein)	−6.97	1.61E-08

**Fig 5:**
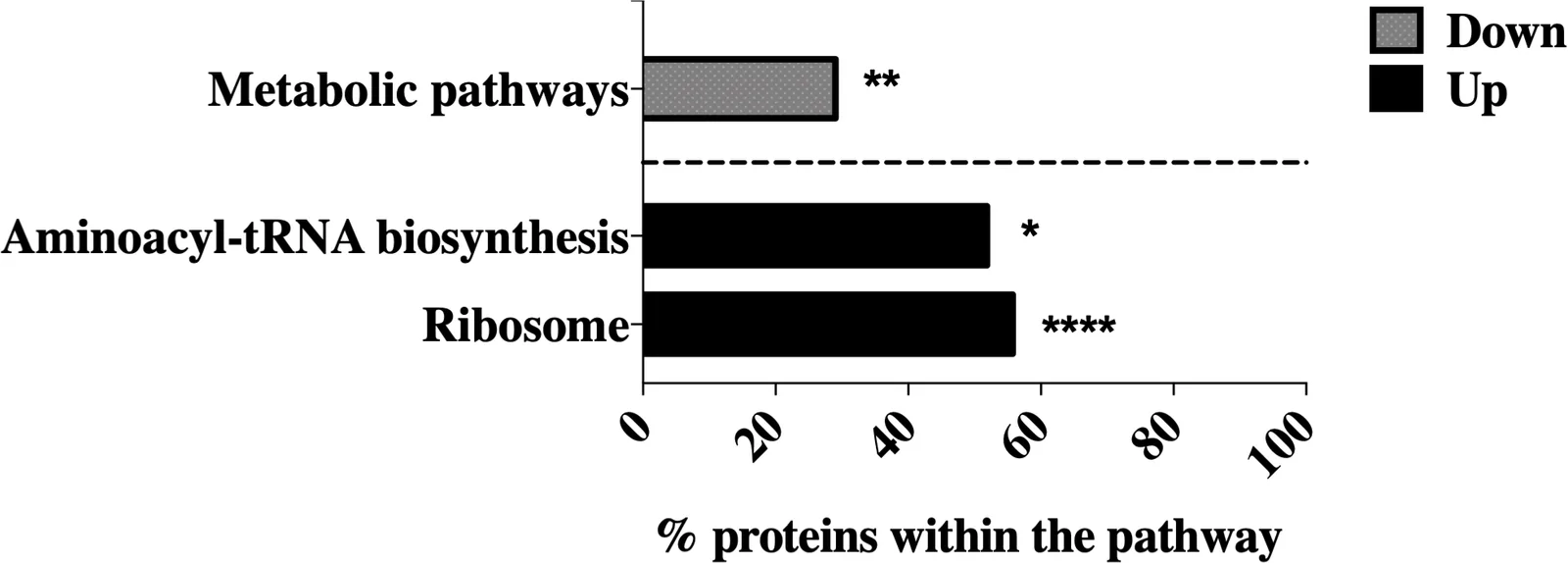
Significantly enriched (FDR corrected *P* ≤ 0.05) KEGG pathways in *G. leopoldii* vs *G. piotii* biofilms. Pathways overrepresented (“Up”) in *G. leopoldii* vs *G. piotii* biofilms are indicated by the black bars, whereas pathways underrepresented (“Down”) in *G. leopoldii* vs *G. piotii* biofilms are indicated by the gray bars. *P ≤ 0.05, **P ≤ 0.01, ****P ≤ 0.0001.

Enrichment analysis using KEGG pathways identified two pathways associated with a ribosomal process and protein synthesis that were significantly overrepresented in *G. leopoldii* vs *G. piotii* biofilms (Fig. 5). Non-specific metabolic pathways were underrepresented in *G. leopoldii* compared to *G. piotii* biofilms (Fig. 5).

### Virulence proteins in *G. leopoldii*, *G. piotii*, and *G. vaginalis* isolate biofilms

**Fig 6:**
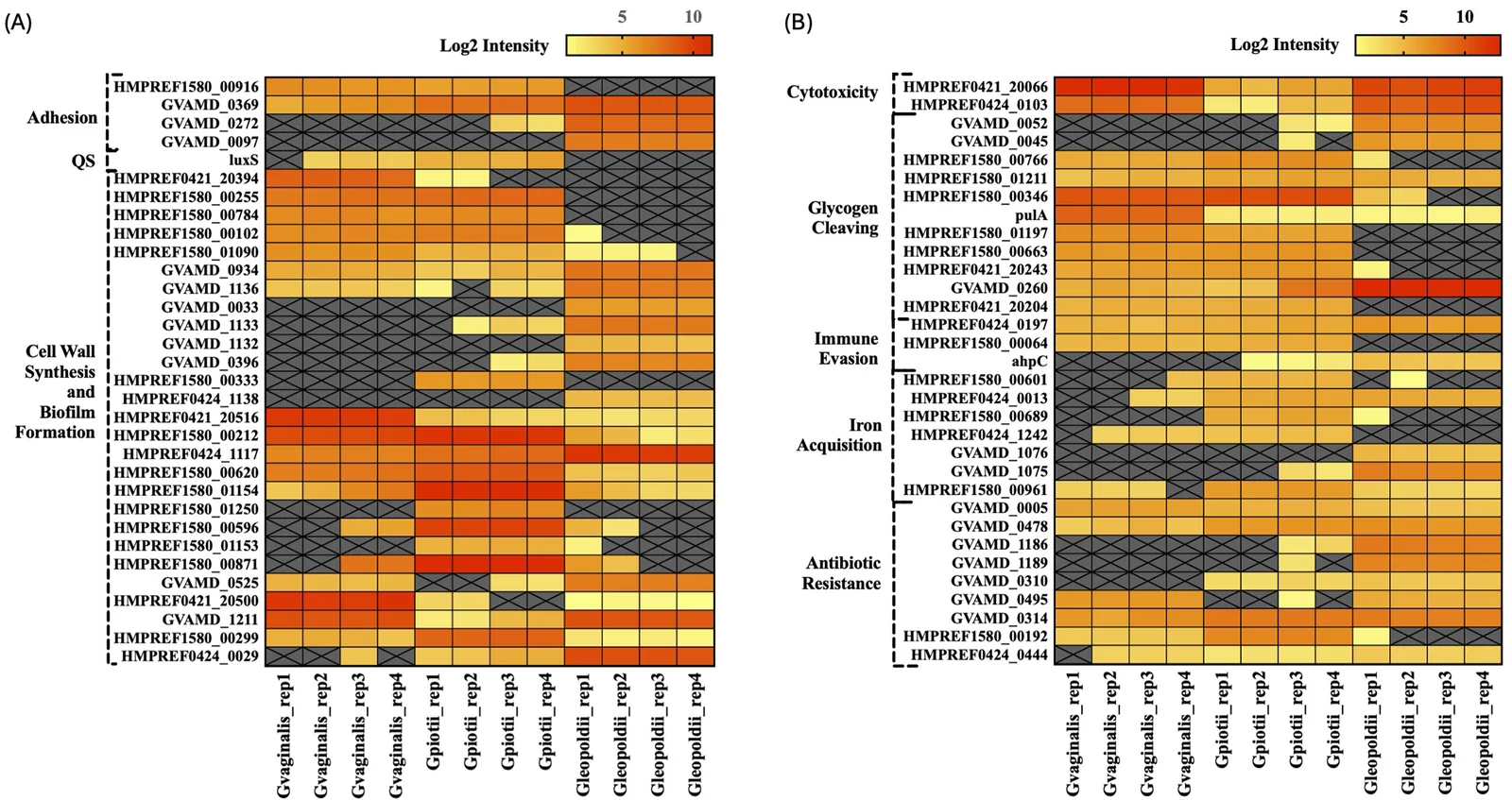
Heatmap of normalized protein signal intensities of virulence proteins predicted to be associated with (**A**) adhesion, quorum sensing (QS), and biofilm formation or (**B**) cytotoxicity, glycogen cleavage, immune evasion, iron acquisition, and antibiotic resistance across *G. vaginalis*, *G. piotii*, and *G. leopoldii* biofilms. Crossed-out gray boxes indicate the protein was not detected in that sample.

**Fig 7:**
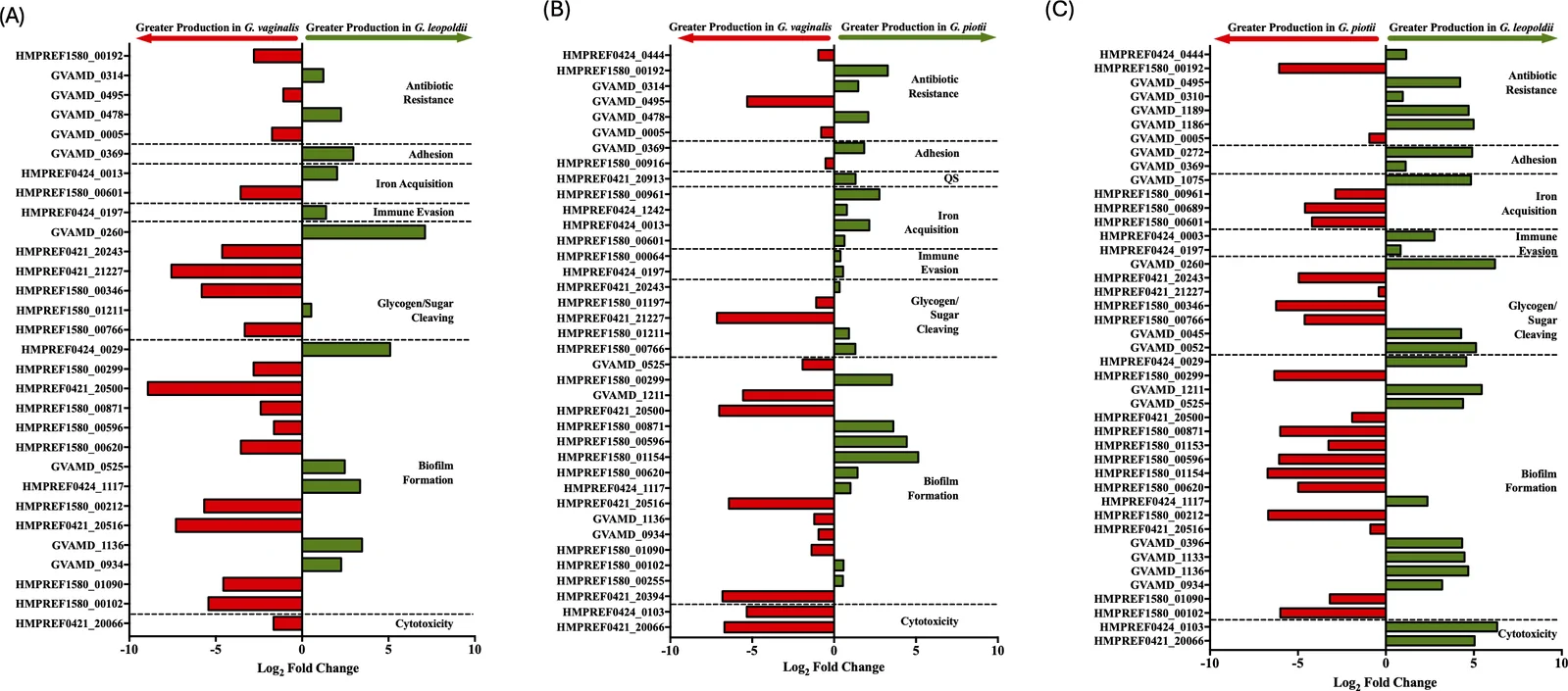
Log_2_ fold changes of significantly (FDR corrected *P* ≤ 0.05) different potential virulence proteins in (**A**) *G. leopoldii* compared to *G. vaginalis* biofilms, (**B**) *G. piotii* compared to *G. vaginalis* biofilms, and (**C**) *G. leopoldii* compared to *G. piotii* biofilms.

Several virulence factors hypothesized to be important for BV pathogenesis have been identified in *Gardnerella* species (25, 26). To look for these virulence factors, a search was performed using a broader database consisting of multiple *Gardnerella* genome references. Virulence proteins were found by either looking for previously published *Gardnerella* virulence proteins (25, 26) or searching the results for proteins with a description of attributed virulence functions (e.g., adhesins, iron acquisition). Several differences were noted in the detection of proteins associated with biofilm formation, as well as bacterial virulence across *G. leopoldii*, *G. piotii*, and *G. vaginalis* biofilms (Fig. 6A and B). Proteins predicted to have glycosyltransferase activity, cell wall synthesis, LPXTG-motif protein cell wall anchor function, or sortase activity were labeled as being involved in biofilm formation (Fig. S1). Statistically significant differences (FDR *P* ≤ 0.05) were noted in the production of proteins predicted to be involved in adhesion and biofilm formation among the assessed biofilms (Fig. 7A and C), highlighting potential mechanistic differences in these processes. A single quorum-sensing associated protein (luxS/HMPREF0421_20913) was identified and detected in this analysis in both *G. piotii* and *G. vaginalis* but not *G. leopoldii* biofilms (Fig. 7A). LuxS encodes for S-ribosylhomocysteine lyase, an autoinducer-2 synthesis protein. This protein was detected at a significantly higher level in *G. piotii* relative to *G. vaginalis* (Fig. 7B). Two cytolysins were identified in our data set, HMPREF0421_20066 and HMPREF0424_0103. Cytolysins were more abundant in *G. vaginalis* biofilms relative to *G. leopoldii* (HMPREF0421_20066) and *G. piotii* (HMPREF0421_20066 and HMPREF0424_0103) biofilms (Fig. 7A and B).

Three immune evasion proteins were identified - HMPREF0424_0197, HMPREF1580_00064, and ahpC/HMPREF0424_0003. HMPREF0424_0197 encodes a macrophage migration inhibitory factor and was found to be produced at significantly higher levels in *G. leopoldii* biofilms relative to both *G. piotii* and *G. vaginalis* biofilms (Fig. 7A and C). HMPREF1580_00064 and ahpC/HMPREF0424_0003 encode alkyl hydroperoxide reductase. This enzyme is involved in the protection of bacteria from oxidative damage (27). HMPREF0424_0003 was produced at significantly greater levels in *G. leopoldii* relative to *G. piotii* biofilms (Fig. 7C), whereas HMPREF1580_00064 was detected at higher levels in *G. piotii* vs *G. vaginalis* (Fig. 7B).

Seven proteins associated with iron acquisition were identified across the studied biofilms (Fig. 6B). Among these iron acquisition proteins, HMPREF1580_00601, HMPREF0424_0013, HMPREF0424_1242, and HMPREF1580_00961 were detected at significantly higher levels in *G. piotii* compared to *G. vaginalis* biofilms (Fig. 7B). Heterogeneity was noted with respect to differences in iron acquisition between *G. leopoldii* and *G. vaginalis* (Fig. 7A), as well as *G. leopoldii* and *G. piotii* (Fig. 7C).

Glycogen is an important nutrient for bacterial growth within the vagina. Glycogen-degrading enzymes, such as alpha-amylases, break the glycogen down into a source that is more readily available (28). Notably, more proteins coding for glycogen-degrading enzymes were identified across *G. vaginalis* and *G. piotii* biofilms compared to *G. leopoldii* biofilms (Fig. 6B). Comparative analysis exposed heterogeneity in differentially produced glycogen-degrading enzymes across *G. leopoldii*, *G. piotii*, and *G. vaginalis* biofilms (Fig. 7A through C). Several potential antibiotic resistance proteins were identified (Table S3), including ABC-type multidrug transporters (GVAMD_0005, GVAMD_0478, GVAMD_1186, GVAMD_1189, GVAMD_0310), ABC-type bacteriocin/antibiotic exporter (GVAMD_0495, GVAMD_0314), and aminoglycoside phosphotransferase domain-containing proteins (HMPREF1580_00192, HMPREF0424_0444). These proteins were identified at higher frequency across *G. leopoldii* biofilms compared to *G. piotii* and *G. vaginalis* biofilms (Fig. 6A and B).

## DISCUSSION

Biofilm formation was examined in representative single isolates of *G. vaginalis*, *G. leopoldii*, and *G. piotii* to better understand species-specific contributions to biofilm formation—an integral step in BV pathogenesis. *G. leopoldii* and *G. piotii* produced biofilms with higher biomass and viability compared to *G. vaginalis*. While growth media may have influenced these results, previous work reported relatively similar biofilm biomass across *Gardnerella* species in both sBHI and NYC III media (20, 29). The discrepancy between these findings and our results may reflect isolate-specific differences, highlighting the paucity of data to date demonstrating the diversity of biofilm formation among clinical isolates. *G. piotii* and *G. leopoldii* produced biofilms with similar viability; however, *G. leopoldii* biofilms exhibited higher biomass. *G. vaginalis* biofilms produced the least biofilm biomass, though with only one log-fold lower CFU count, possibly reflecting differences in extracellular matrix quantity and/or composition. The robust biofilm formation observed in the *G. leopoldii* isolate may contribute to the reported association between high abundance of *G. swidsinskii*/*G. leopoldii* measured via qPCR in a longitudinal study and increased BV recurrence following standard metronidazole treatment (30). Proteins associated with DNA replication were more abundant in *G. vaginalis*—suggestive of a more active planktonic-preferring phenotype, which correlates with its lower biofilm formation in this study. This study successfully cultured bacteria from all three species’ biofilms, contrasting with findings reported by Castro et al., who used the same culture medium and incubation times but a different *G. vaginalis* isolate (ATCC 14018) that, despite high biomass and viability, resulted in no culturable bacteria (31). The *G. vaginalis* isolate used here (ATCC 49145) shares >96% sequence similarity in both *cpn*60 and 16S rRNA regions (20). Nonetheless, ATCC 49145 was reported to produce higher biofilm biomass than ATCC 14018 in NYC III broth (20), suggesting that the observed discrepancies between studies may be attributable to strain-specific differences.

Significant variability in proteomes and predicted virulence proteins was observed across *G. leopoldii*, *G. piotii*, and *G. vaginalis* isolate biofilms. These differences in protein abundance could reflect either variation in gene presence/absence among strains or differences in gene expression levels. Cysteine synthase (CysK) was among the most overrepresented proteins in the higher biomass *G. leopoldii* and *G. piotii* biofilms compared to the lower biomass *G. vaginalis* biofilms. CysK is involved in the production of cysteine and acetate (32) but may also perform additional, currently uncharacterized (moonlighting) functions in *Gardnerella* species (33, 34). Previously, CysK was shown to promote biofilm formation in *Vibrio fischeri* (35) and was significantly upregulated in multiple *Gardnerella* species’ large colony variants (hypothesized to contribute to biofilm formation) (36). Although cysteine concentrations were not measured here, previous work showed that cysteine is found at lower concentrations in BV-positive samples (37, 38). The relationship between these observations and the present findings remains unclear; however, one explanation may be due to the consumption of cysteine by BV-associated bacteria. Indeed, previous *in vitro* work showed that amino acids produced by *Gardnerella* are consumed by *P. bivia*, which in turn produces ammonia that can further enhance *Gardnerella* growth (39). *Lactobacillus iners*, which frequently increases in abundance following standard BV treatment (40–42), is also dependent on cysteine availability (38). Whether CysK contributes directly to biofilm formation in the isolates examined here, either through cysteine synthesis or an alternative function, was not tested. If a functional role for CysK in biofilm formation is confirmed, it could represent a potential target for strategies aimed at disrupting *Gardnerella* biofilms.

The pathogenesis of BV likely begins with the displacement of optimal lactobacilli, followed by biofilm initiation by virulent *Gardnerella* species, which promote the growth of other BV-associated bacteria (18). Colonization by *Gardnerella* species, which are facultative anaerobes, therefore requires at a minimum (i) evasion of the local immune response, (ii) ability to effectively utilize host nutrients, and (iii) adhesion to vaginal epithelial cells. Here, significant differences were identified in the production of proteins that may play a role in immune evasion, including cytolysins (43–45), two alkyl hydroperoxide reductases, and a predicted macrophage migration inhibitory factor. Cytolysins were detected at significantly higher levels in the thinner *G. vaginalis* biofilms compared to the robust biofilms produced by *G. leopoldii* and *G. piotii*. This pattern may reflect context-dependent strategies: in the planktonic state, higher cytolysin levels could help directly modulate or evade host immune cells via direct cell lysis, whereas in robust biofilms, reduced cytolysin production could minimize immune activation and allow biofilm persistence, as previously described (26, 46). Alkyl hydroperoxide reductases (antioxidative enzymes) and a predicted macrophage migration inhibitory factor were detected across all the studied *Gardnerella* biofilms; however, these were detected at a significantly higher level in *G. leopoldii* relative to *G. piotii* and *G. vaginalis* biofilms. Intriguingly, previous *in vitro* work using different *Gardnerella* isolates (not grown as biofilms) showed that the tested isolates induced reactive oxygen species production in macrophages (47). The possession of alkyl hydroperoxide reductases and macrophage inhibitory factors may thus confer an ecological advantage (48, 49).

Glycogen is an important source of carbohydrates for vaginal microbes (50). Recent work by Bhandari et al. demonstrated activity of glycogen-degrading enzymes across all *Gardnerella* species (28). Here, fewer glycogen-degrading enzymes were identified in *G. leopoldii* biofilms compared to *G. piotii* and *G. vaginalis*, although those detected were at a high level. It is unclear whether these biofilms differ in their effectiveness in breaking down glycogen, as this was not assessed here. Significant variations were noted with respect to the adhesion proteins detected across the studied isolates. This could suggest that some of these adhesion proteins are more effective, given the observed differences in biofilm biomass and viability. Although a previous study utilizing electron microscopy did not detect pili on the same *G. leopoldii* isolate used in our study (51), our analysis did identify adhesion proteins. Proteins previously identified as having potential roles in *Gardnerella* biofilm formation include glycosyltransferases, sortases, and proteins carrying LPXTG motifs (25). *G. vaginalis*, *G. piotii*, and *G. leopoldii* biofilms were all found to produce these proteins, although at different levels. These findings emphasize that biofilm formation in *Gardnerella* is not driven by a single set of conserved proteins but rather by flexible, strain-dependent mechanisms that likely impact community dynamics in the vaginal environment.

Several limitations are implicated in this study. First, only a single isolate was examined for each species, limiting the ability to generalize findings. Only three of the six described *Gardnerella* species were investigated, limiting the scope of our conclusions (22, 52). This study did not assess the proportion of extracellular biofilm matrix versus bacterial cells within the biofilms. In addition, this work only examined biofilm formation within a simplified *in vitro* environment that does not fully capture the complexity of the vaginal niche. Most of the entries in UniProt for *Gardnerella* are unreviewed, resulting in many of the proteins detected being uncharacterized. Efforts are urgently needed to improve the characterization of *Gardnerella* (and other BV-associated taxa) proteins to enhance the application of proteomics to the study of *Gardnerella* and the vaginal microbiome.

These findings can be considered in the context of current hypothetical models of BV pathogenesis, in which *Gardnerella* species initiate biofilm formation and facilitate colonization by other BV-associated bacteria (18). With respect to the isolates tested here, our data suggest species-specific functional differences, with *G. leopoldii* and *G. piotii* producing more robust biofilms and higher levels of certain proteins associated with biofilm formation and immune evasion compared to *G. vaginalis*. While these results indicate potential differences in virulence-related traits *in vitro*, we cannot directly conclude that one species is more virulent *in vivo*. Future studies incorporating host interactions are needed to clarify the contributions of each *Gardnerella* species to BV development and persistence.

In summary, this study suggests *G. leopoldii* and *G. piotii* may be better equipped for the initial establishment of the biofilm state, given the more robust biofilms produced. Immune evasion proteins, which were expressed at the highest level in *G. leopoldii*, further shed light on the ecological advantage this species may possess. Given the emerging appreciation of *Gardnerella* heterogeneity, these findings provide insight into functional differences among these newly established species and underscore the importance of species-level resolution in studies of *Gardnerella* biofilms.

## MATERIALS AND METHODS

### Bacterial isolates, growth conditions, and quantification

*Gardnerella* strain AMD (NR-50514) and strain JCP8070 (HM-1113) were obtained from BEI Resources (NIAID, NIH, as part of the Human Microbiome Project [HMP]). *Gardnerella* strain 49145 was obtained from the American Type Culture Collection (ATCC). This work focused on *G. leopoldii* (AMD), *G. piotii* (JCP8070), and *G. vaginalis* (ATCC 49145) because these isolates represent at least one taxon from the established *Gardnerella* subgroups/clades, have been characterized to some extent, and were commercially available. All isolates were grown on Columbia agar supplemented with 5% sheep blood (23 g/L special peptone, 1 g/L special starch, 5 g/L NaCl, 10 g/L agar, 5% sheep blood) for 48 h. Isolated colonies were streaked on Columbia agar supplemented with 5% sheep blood plates and incubated for an additional 48 h.

Colonies of similar morphology were used to inoculate overnight cultures in NYC III (ATCC 1685) broth (4 g/L HEPES, 15 g/L proteose peptone #3, 5 g/L NaCl, 0.5% glucose, 6.25 g/L yeast extract, 10% horse serum). NYC III broth was chosen because it supports robust planktonic and biofilm growth across *Gardnerella* species, as previously demonstrated by Rosca et al. (29). *Gardnerella* isolates were grown throughout this study at 37°C under anaerobic conditions using GasPak EZ containers (BD-Canada, Mississauga, ON, Canada) with sachets per the manufacturer’s protocol. Growth curves were constructed to determine the growth properties of the *Gardnerella* isolates in NYC III broth and to estimate CFU/mL from OD_600_ measurements for biofilm experiments (Fig. S1).

The genomospecies classification of each isolate used was confirmed using *cpn*60 sequencing (Table 4). Briefly, DNA was extracted using the DNeasy Blood and Tissue Kit (QIAGEN) with the manufacturer’s enzymatic lysis step for Gram-positive bacteria. The *cpn*60 gene was amplified using primers H729 and H1134 (Table S4) with 5 µL of template DNA added to 45 µL of PCR mastermix containing 1× PCR buffer II, 0.2 mM dNTP mix, 2 mM MgCl_2_, 0.4 µM of each primer, and 1.25 units of AmpliTaq Gold DNA polymerase. PCR was then run on a thermocycler set to initial denaturation at 95°C for 10 min, followed by 40 cycles of 95°C for 30 s, 50°C for 30 s, 72°C for 45 s, and a final extension step at 72°C for 10 min. Purified amplicons were subjected to standard Sanger sequencing. Sequences were assigned to *Gardnerella* genomospecies using the chaperonin database (cpndb.ca).

**TABLE 4:** Subgroup and genomospecies nomenclature of the *Gardnerella* isolates included in this study, as confirmed by *cpn*60 direct isolate sequencing

*Gardnerella* isolate	Subgroup	Genomospecies nomenclature
AMD	A	*G. leopoldii* genomospecies 5
JCP8070	B	*G. piotii* genomospecies 4
ATCC 49145	C	*G. vaginalis* genomospecies 1

### Biofilm growth and harvesting

Overnight cultures of each isolate were subcultured and grown to mid-log phase, then diluted to ~10^7^ CFU/mL in NYC III broth. Inoculum concentrations were confirmed by spot plating using three technical replicates. Fifteen milliliters of the bacterial suspension were added to T75 tissue culture flasks: four biological replicates for lysate preparation (for proteomics), and three biological replicates for biofilm biomass quantification. A negative control flask was prepared with 15 mL of sterile NYC III broth to monitor for contamination. T75 flasks were used to generate sufficient biofilm biomass for downstream proteomic analyses.

Flasks were agitated manually to ensure even dispersion of the bacteria in the flask and grown in GasPak EZ anaerobic containers at 37°C. After 24 h, planktonic cells were removed, and each flask was washed with 5 mL of NYC III broth to remove any remaining planktonic bacteria. Fresh 15 mL NYC III broth was added to the flasks, which were gently agitated manually and incubated for an additional 24 h at 37°C to allow further biofilm accumulation for downstream analysis (26).

At 48 h, the media was removed, and biofilms were washed twice with 5 mL NYC III broth to remove residual planktonic bacteria. For downstream proteomic analysis, 10 mL NYC III broth was added to each flask, and biofilms were scraped with a cell scraper and collected into a sterile tube. Flasks were rinsed with an additional 5 mL NYC III broth to transfer any remaining biofilm to the collection tube.

The collected biofilm was then mixed to resuspend, and 80 µL were set aside for spot plating to determine viable bacterial counts inside the biofilms. The remaining biofilm in the collection tube was then centrifuged at 3,750 rpm at 4°C for 30 min and left at 4°C for an additional 30 min. The supernatant was removed, and the pellet was resuspended in 1 mL of 0.9% saline and transferred to a microfuge tube and centrifuged at 5,000 rcf for 3 min at 4°C. The supernatant was removed, and the pellet was washed twice in 1 mL of 0.9% saline to remove contaminating media proteins.

### Biofilm quantification

Viable bacterial counts within biofilms were determined by spot plating aliquots of resuspended biofilms on Columbia agar supplemented with 5% sheep blood in duplicate. Total biofilm biomass was assessed using crystal violet staining (53) with volume adjustments due to the use of T75 flasks.

For quantification of biofilm biomass, the media was removed from each of the quantification and negative control flasks, and each biofilm was then rinsed twice with 5 mL NYC III broth, followed by a rinse with 10 mL 0.9% saline solution. Seven milliliters of 1% crystal violet solution was added to each flask, followed by a 5-10 min incubation at room temperature. The crystal violet was then removed from the flask, and the biofilms were washed twice with 50 mL of distilled water to remove any unbound crystal violet.

Any excess liquid was then removed carefully with a serological pipette, and the flasks were allowed to dry overnight in a biosafety cabinet. The next day, the biofilms were photographed using a smartphone camera (iPhone 7) without flash or magnification under the biosafety cabinet’s built-in lighting. Following this, the crystal violet was solubilized using 10 mL of 33% acetic acid with a 10-15 min incubation at room temperature. The contents of the flask were then mixed thoroughly and transferred to a collection tube, mixed again, and OD_595_ was measured using the Synergy H1 spectrophotometer (in triplicate).

### Protein isolation

Detergent-based lysis combined with mechanical disruption was used to enhance protein recovery (54). The biofilm pellet was resuspended in 1,000 µL of lysis buffer containing 2% sodium dodecyl sulfate (SDS), 100 mM dithiothreitol, and 50 mM HEPES (pH 8.3) prepared in mass spectrometry-grade water. The suspension was transferred to tubes containing approximately 200 µL of 0.1 mm spherical glass disruptor beads (Scientific Industries Inc., Bohemia, NY, USA) and subjected to mechanical disruption using the Bead-Ruptor Elite (Omni International Inc.) for six cycles of 6.3 m/s for 45 s with a 1-min dwell time.

The tubes were placed on ice and centrifuged at 20,800 rcf for 10 min at 4°C to pellet debris and beads. The supernatant was transferred to a fresh tube, and residual material was extracted with an additional 1,000 µL of lysis buffer, followed by centrifugation under the same conditions. Tubes in which the glass beads did not settle were subjected to an additional centrifugation step, and the supernatant was carefully collected without disturbing the beads. SDS-PAGE confirmed protein extraction.

### Proteomics sample preparation

One hundred micrograms of each sample was made to 5% SDS to a total volume of 100 µL. The samples were processed using an on-filter digestion protocol using S-Trap mini spin columns (Protifi, Fairport, NY, USA) as per the manufacturer’s protocol. The samples were digested with trypsin (Pierce Trypsin, Protease). The digested samples were concentrated to near dryness using a vacuum centrifuge, then labeled with TMT 16plex Isobaric Label Reagent Set (Thermo Fisher Scientific) as per the manufacturer’s protocol. Labeled samples for each TMT experiment were mixed one-to-one. Mixed TMT samples were fractionated into 12 fractions by high-pH C_18_ reversed-phase liquid chromatography using an Agilent 1260 Infinity liquid chromatography system (Agilent Technologies) equipped with an XBridge C18 column (2.1 × 100 mm, Waters). Each fraction was concentrated to near-dryness using vacuum centrifugation, resuspended in 80 µL of buffer A (2% acetonitrile, 0.1% formic acid).

### Mass spectrometry

Each fractionated sample was analyzed by liquid chromatography-tandem mass spectrometry using a nano-flow Easy-nLC 1200 connected in-line to an Orbitrap Fusion Lumos mass spectrometer (Thermo Fisher Scientific). The samples were run through a 50-cm Easy-Spray C18 column (Thermo Fisher Scientific) with a 120-min gradient. The following settings were used for data acquisition. The precursor scans were acquired in positive mode in the Orbitrap with a range of 375–1,500 *m*/*z* at 120,000 resolution. Fragmentation scans were done in the ion trap using CID fixed at a 35% collision energy with a 1.2 *m*/*z* isolation window, fragmenting as many targets as possible in a 2-s window. Each of the targets was also fragmented again using MS3 with HCD set to 65% normalized collision energy, five dependent scans, and analyzed in the Orbitrap with a resolution set to 50,000.

### Data analysis

Spectral data were processed using the Proteome Discoverer version 2.4 software (Thermo Fisher Scientific), using the Mascot search engine for peptide sequencing. The search database was the *G. vaginalis* HMPREF3204 genome (BioProject PRJNA272096; assembly GCA_001546445.1, sometimes annotated as *G. pickettii*). This was selected as it is the database of choice for STRINGdb for functional annotation of *Gardnerella* proteins. Protein identification and quantification were performed using the built-in Proteome Discoverer templates for processing and consensus workflows. Default settings were used apart from normalization and intensity scaling, which were disabled. For a targeted search for *Gardnerella* virulence proteins, a broader database was created using publicly available *Gardnerella* reference sequences, including AMD (taxid 682147), ATCC 14019 (taxid 525284), JCP8070 (taxid 1261065), and HMP reference strains HMPREF0421 (BioProject PRJNA31473) and HMPREF0424 (BioProject PRJNA31001). These sequences were downloaded on 5 August 2022.

Protein intensities were log2-transformed and median-normalized using the median_normalization function in the proDA R package. Normalized protein levels were averaged across all four biological replicates, and the values were compared among *Gardnerella* pairs (*G. leopoldii* vs *G. vaginalis*, *G. piotii* vs *G. vaginalis*, and *G. leopoldii* vs *G. piotii*) using the limma package (55) on R (version: 4.3.1). Functional enrichment analysis was performed using STRINGdb (56) (https://string-db.org/). Heatmaps and figures were generated using R (version 4.3.1) and GraphPad Prism 7.

## Data Availability

All associated mass spectrometry data are available for review on MaSSIVE MSV000097483 (proteome xchange PXD062480). All genome sequences used to construct the custom *Gardnerella* database are publicly available in NCBI under taxids 682147 (AMD), 525284 (ATCC 14019), 1261065 (JCP8070), and BioProjects PRJNA31473 (HMPREF0421), and PRJNA31001 (HMPREF0424). All sequences were accessed on 5 August 2022.

## SUPPLEMENTAL MATERIAL

Supplemental figures and tables jb.00416-25-s0001.pdf Figure S1 and Tables S1 to S4.

ASM does not own the copyrights to Supplemental Material that may be linked to, or accessed through, an article. The authors have granted ASM a non-exclusive, world-wide license to publish the Supplemental Material files. Please contact the corresponding author directly for reuse.
