# Supplementary material for: Comparative proteomics reveal striking differences in biofilm formation and virulence potential of Gardnerella leopoldii, G. piotii, and G. vaginalis isolates
Source: J Bacteriol. 2026 Jul 20;208(8):e00416-25. doi: 10.1128/jb.00416-25 (PMC13492103; doi:10.1128/jb.00416-25)
Supplement: Supplementary file 1 [file jb.00416-25-s0001.pdf]

Table S1. Seeding concentrations and viability of studied *Gardnerella* spp. biofilms.

	***G. leopoldii* AMD**	***G. piotii* JCP8070**	***G. vaginalis* ATCC 49145**
**Seeding concentration (CFU/mL)**	2.30×10^7	2.90×10^7	2.10×10^7
**Biofilm viability (CFU) at 48 hours (Range)**	2.85×10^9 (2.1×10^9 - 3.68×10^9)	1.63×10^9 (1.35×10^9 - 2.18×10^9)	1.33×10^8 (8.25×10^7 - 1.73×10^8)

Note: the same seeding concentration was used across all biological replicates.

Table S2. Table of UniProt accession numbers of the unique and shared proteins detected across the studied *G. leopoldii*, *G. piotii*, *G. vaginalis* biofilms.

Taxa	Total	UniProt Accession Number
Detected in all: *G. leopoldii G. piotii G. vaginalis*	511	A0A133NFY1 A0A133NHA5 A0A133NJ90 A0A0J8FC58<br>A0A133NSV2 A0A133NJ33 A0A133NKR6 A0A133NJF9<br>A0A133NIX4 A0A133NL55 A0A133NIT8 A0A133NG53<br>A0A133NIF6 A0A133NME0 A0A133NFV3 A0A133NIA7<br>A0A133NHE6 A0A133NJ06 A0A133NG23 A0A133NK72<br>A0A133NJH0 A0A133NLT6 A0A133NJH7 A0A133NIC9<br>A0A133NHQ9 A0A133NHT6 A0A133NL56 A0A133NMT9<br>A0A133NLQ7 A0A133NML5 A0A133NGX9 A0A133NK87<br>A0A133NLQ6 A0A133NLA1 A0A133NPW9 A0A133NGE8<br>A0A133NIA0 A0A133NKL5 A0A133NJF7 A0A133NLI0<br>A0A133NGJ7 A0A133NKP4 A0A133NKM5 A0A133NJ46<br>A0A0J8I4Q1 A0A133NIV8 A0A0J8F960 A0A133NG15<br>A0A133NLH2 A0A0J8FD71 A0A133NHS9 A0A133NFZ2<br>A0A133NLI5 A0A133NI51 A0A133NIJ3 A0A133NL74<br>A0A133NHZ6 A0A133NJC0 A0A133NGI6 A0A133NKL3<br>A0A133NL19 A0A133NLM9 A0A133NJD1 A0A133NJI4<br>A0A133NMM7 A0A133NI70 A0A135Z8W7 A0A133NK51<br>A0A133NGZ2 A0A133NI84 A0A133NHE4 A0A133NLU2<br>A0A133NL67 A0A133NHX5 A0A133NJC3 A0A133NPU9<br>A0A0J8FD80 A0A133NKA6 A0A133NFW2 A0A133NIX0<br>A0A133NFV5 A0A133NG36 A0A133NFR5 A0A133NJ32<br>A0A133NGZ7 A0A133NGE1 A0A133NKZ6 A0A0J8FDH6<br>A0A133NG75 A0A133NMS5 A0A133NFR0 A0A133NG58<br>A0A133NI72 A0A133NG35 A0A133NGA6 A0A133NH12<br>A0A133NL85 A0A133NLJ1 A0A133NHZ9 A0A133NKY5<br>A0A133NG97 A0A0J8FEX7 A0A133NHI5 A0A133NJ07<br>A0A133NFX6 A0A133NMT3 A0A0J8F821 A0A133NGN2<br>A0A133NGX1 A0A133NG26 A0A133NL82 A0A133NGH0<br>A0A133NLC5 A0A133NHV7 A0A133NHW4 A0A133NHD2<br>A0A133NLT5 A0A133NK40 A0A133NL93 A0A133NGB4<br>A0A133NH64 A0A133NFV0 A0A133NIJ2 A0A133NHY3<br>A0A133NM08 A0A133NL24 A0A133NI48 A0A133NGM2<br>A0A133NHG1 A0A133NL57 A0A133NFW9 A0A133NJI3<br>A0A133NG65 A0A133NIG0 A0A133NI40 A0A133NI42<br>A0A133NGB1 A0A133NKZ1 A0A133NJ40 A0A133NHC7<br>A0A133NHZ0 A0A133NGK7 A0A133NGH8 A0A133NLQ9<br>A0A133NGP2 A0A133NH87 A0A133NHE3 A0A0J8F815<br>A0A133NFX2 A0A133NKJ6 A0A133NI82 A0A133NFU3<br>A0A133NLT1 A0A133NHH5 A0A133NIB8 A0A133NKK4<br>A0A133NG77 A0A133NIL1 A0A133NHV3 A0A133NHZ8

A0A133NIU2 A0A133NIP4 A0A133NJ57 A0A133NI90 A0A133NIY8 A0A133NK57 A0A133NHX6 A0A133NI71 A0A133NHB7 A0A133NHR9 A0A133NL32 A0A133NG62 A0A133NI79 A0A133NL89 A0A133NI62 A0A133NFY6 A0A133NI20 A0A133NI98 A0A0J8I526 A0A133NG81 A0A133NFW8 A0A133NJ04 A0A133NJ28 A0A133NK25 A0A133NMC3 A0A133NL81 A0A133NGV6 A0A133NL05 A0A133NKP9 A0A133NH58 A0A133NGN1 A0A133NJK0 A0A133NHA3 A0A133NGJ0 A0A133NLG5 A0A133NI69 A0A133NJ27 A0A133NME6 A0A133NL58 A0A133NG93 A0A133NIF7 A0A133NL42 A0A133NIZ1 A0A133NGE7 A0A133NIH0 A0A133NG89 A0A133NHH4 A0A133NFM4 A0A133NGL9 A0A133NNG0 A0A133NK46 A0A133NGA8 A0A133NI13 A0A133NIB5 A0A133NGW8 A0A133NIG6 A0A133NGK8 A0A133NKM8 A0A133NG64 A0A133NIX2 A0A133NK54 A0A0J8F8W6 A0A133NIT6 A0A133NI30 A0A133NKN2 A0A133NJF6 A0A133NKM7 A0A133NI52 A0A133NGK0 A0A133NHR1 A0A133NGQ4 A0A133NJA2 A0A133NIR2 A0A133NHX0 A0A133NKP3 A0A133NKQ1 A0A133NHW1 A0A0J8FC29 A0A133NGG8 A0A133NG63 A0A133NL65 A0A133NJ10 A0A133NL13 A0A133NMD9 A0A133NJ01 A0A133NID8 A0A133NLN0 A0A133NL06 A0A133NGB9 A0A133NFT7 A0A133NGA3 A0A133NI99 A0A133NLQ1 A0A133NLV0 A0A133NIT3 A0A133NHC6 A0A133NKM0 A0A133NJH4 A0A0J8I4Q5 A0A133NI14 A0A133NIT2 A0A0J8F7W4 A0A133NJF5 A0A133NGB5 A0A0J8I4N8 A0A133NIH4 A0A133NI68 A0A133NHG7 A0A133NG39 A0A133NH75 A0A133NHD7 A0A133NG79 A0A133NL70 A0A133NH28 A0A133NLM5 A0A133NGL1 A0A133NIV0 A0A133NGV4 A0A133NGE6 A0A133NJC7 A0A133NJ05 A0A133NFU5 A0A133NHE9 A0A133NJC6 A0A133NLB5 A0A133NPY2 A0A133NL78 A0A133NLU1 A0A133NLH6 A0A133NKN9 A0A133NII1 A0A133NMS3 A0A133NH42 A0A133NMU1 A0A133NKP8 A0A133NJ64 A0A133NLI8 A0A133NHY1 A0A133NG72 A0A133NL14 A0A0J8F7W1 A0A133NJA6 A0A133NL61 A0A133NI25 A0A133NGB8 A0A133NKX5 A0A133NGJ3 A0A133NL08 A0A133NHE2 A0A133NHR2 A0A133NFR2 A0A133NIE9 A0A133NK28 A0A133NI44 A0A133NI04 A0A133NLQ3 A0A0J8F7R0 A0A133NK83 A0A133NL38 A0A133NLV1 A0A133NK60 A0A133NGL0 A0A133NFZ4 A0A133NK53 A0A133NIB3 A0A133NJH2 A0A133NL30 A0A133NQ07 A0A133NK80 A0A133NI26 A0A133NFZ0 A0A133NL98 A0A133NGE4 A0A0J8FAF6 A0A133NMS9 A0A133NHD8 A0A133NI41 A0A133NFY3 A0A133NMT5 A0A133NJH5 A0A133NGD5 A0A133NLT2 A0A133NMF3 A0A133NI15

A0A133NGJ1 A0A133NKN7 A0A133NL73 A0A133NH47 A0A133NH91 A0A133NL53 A0A133NI34 A0A133NME2 A0A133NG80 A0A133NFS2 A0A133NH79 A0A133NFX4 A0A133NHT0 A0A133NFV1 A0A0J8F755 A0A133NGG1 A0A133NHE1 A0A133NGC9 A0A133NLW3 A0A133NIC7 A0A133NIR0 A0A133NFS7 A0A133NKK1 A0A133NKP0 A0A133NGC4 A0A133NG88 A0A133NJ26 A0A133NMY0 A0A0J8F7W7 A0A133NL52 A0A0J8I3N6 A0A0J8FD92 A0A133NI27 A0A133NIG4 A0A133NI87 A0A133NJ54 A0A133NI74 A0A133NHY2 A0A133NK55 A0A133NJ66 A0A133NJ47 A0A133NGI2 A0A133NKY6 A0A133NK41 A0A133NL68 A0A133NLI3 A0A133NIK3 A0A133NI95 A0A133NI46 A0A133NG84 A0A133NK21 A0A133NLU6 A0A133NI47 A0A133NIV2 A0A133NIU7 A0A133NFU1 A0A133NJ63 A0A0J8I4T2 A0A133NLJ4 A0A133NMP7 A0A133NKW9 A0A133NL41 A0A133NMU7 A0A133NKQ9 A0A133NJ96 A0A133NL01 A0A133NL96 A0A133NJB6 A0A133NII3 A0A133NJ73 A0A133NLT4 A0A133NJ22 A0A133NKX7 A0A133NIM0 A0A133NHH0 A0A0J8FCQ9 A0A133NLP2 A0A133NK73 A0A133NH86 A0A133NHA9 A0A133NL77 A0A133NIG1 A0A133NGB3 A0A133NFT2 A0A133NIT0 A0A133NL51 A0A133NIE1 A0A133NK43 A0A133NNS1 A0A133NI06 A0A133NIA5 A0A133NHI9 A0A133NH45 A0A133NMU3 A0A133NG29 A0A133NJH1 A0A133NHR4 A0A0J8FCT6 A0A133NIC0 A0A133NMS1 A0A133NFX7 A0A133NI32 A0A133NLY5 A0A133NHI0 A0A133NFV4 A0A133NKK5 A0A133NHF9 A0A133NLA4 A0A133NIC3 A0A133NKK6 A0A133NL21 A0A133NHB0 A0A133NIH8 A0A133NFX8 A0A133NJB1 A0A133NJ95 A0A133NG82 A0A133NK99 A0A133NHB8 A0A133NGB6 A0A133NK48 A0A133NFS8 A0A133NI76 A0A133NHX3 A0A133NL18 A0A133NPB9 A0A133NME9 A0A133NI67 A0A133NHC4 A0A133NII2 A0A133NG33 A0A133NK22 A0A133NIS7 A0A133NLQ2 A0A133NL22 A0A133NG67 A0A133NMU6 A0A133NGP3 A0A133NGK9 A0A133NFU6 A0A133NI10 A0A133NKZ5 A0A133NIP7 A0A133NMD6 A0A133NKP7 A0A133NHV5 A0A133NMG0 A0A133NHD5 A0A133NL84 A0A133NJ80 A0A133NGD4 A0A133NFV9 A0A133NLI9 A0A133NL87 A0A133NI31 A0A133NIG2 A0A133NL26 A0A133NFU7 A0A133NK50 A0A133NGM0 A0A133NKX8 A0A133NHY5 A0A133NLU9 A0A133NL60 A0A133NG28 A0A133NIA6 A0A133NHZ2

Detected in both *G. piotii* and *G. vaginalis*	96	A0A133NGK1 A0A133NMF6 A0A133NHF3 A0A133NGW3 A0A133NL46 A0A133NJ02 A0A133NK71 A0A133NHG0 A0A133NHV9 A0A133NJA3 A0A133NHW2 A0A133NHW9 A0A133NHF1 A0A133NLI4 A0A133NH50 A0A133NGF2 A0A133NFX0 A0A133NIF9 A0A133NGW1 A0A133NL28 A0A133NHX2 A0A133NIF0 A0A133NLI1 A0A133NFZ7 A0A133NJ84 A0A133NKQ6 A0A133NLH4 A0A133NHY4 A0A133NIS8 A0A133NKJ0 A0A133NH67 A0A133NIQ8 A0A133NHF5 A0A133NG51 A0A133NIW5 A0A133NJ93 A0A133NML1 A0A133NLF4 A0A133NFZ5 A0A133NFS3 A0A133NJ99 A0A133NLP6 A0A133NLB0 A0A133NLB6 A0A133NLH1 A0A133NL59 A0A133NFQ0 A0A133NGD7 A0A133NHK8 A0A133NJ03 A0A133NKM9 A0A133NGI3 A0A133NK27 A0A133NKL1 A0A133NG44 A0A133NKN1 A0A133NKK2 A0A133NL79 A0A133NGA2 A0A133NFV7 A0A133NMM5 A0A133NKJ1 A0A133NKM2 A0A0J8I6Q0 A0A133NLS9 A0A133NJ44 A0A133NGF9 A0A133NKX6 A0A133NI23 A0A133NG41 A0A133NG16 A0A133NGD1 A0A133NK37 A0A133NKI8 A0A133NHE0 A0A133NGL8 A0A133NGW2 A0A133NG74 A0A133NIR5 A0A133NH63 A0A133NLD0 A0A133NHG4 A0A133NG05 A0A133NJ09 A0A133NLS3 A0A133NHV8 A0A133NL02 A0A133NJ39 A0A133NIZ5 A0A133NGA7 A0A133NK42 A0A133NIE6 A0A133NKJ4 A0A133NKL8 A0A133NKJ9 A0A133NMI6
Detected in both *G. leopoldii* and *G. vaginalis*	3	A0A133NGI5 A0A133NLV2 A0A133NJC5
Detected in both *G. leopoldii* and *G. piotii*	64	A0A133NHF2 A0A133NKL0 A0A133NI08 A0A133NFV6 A0A133NI83 A0A133NMT2 A0A133NI78 A0A133NG95 A0A133NLU8 A0A0J8FED4 A0A133NMQ5 A0A133NH94 A0A133NI97 A0A133NHV2 A0A133NJB3 A0A133NIB6 A0A133NMQ4 A0A133NIC4 A0A133NHG5 A0A0J8FAF5 A0A133NL44 A0A133NGI0 A0A133NIG5 A0A133NG21 A0A133NFU2 A0A133NID6 A0A133NFT9 A0A133NHC8 A0A133NLT7 A0A133NKZ0 A0A133NLU4 A0A133NHC9 A0A133NFW7 A0A133NGN3 A0A133NFT3 A0A133NMJ1 A0A133NFW3 A0A133NIA3 A0A133NJB7 A0A133NME1 A0A133NKK7 A0A133NK35 A0A133NMU4 A0A133NMP3 A0A133NIW3 A0A133NHJ4 A0A133NGY7 A0A133NKN0 A0A133NK68 A0A133NMT6 A0A133NGK3 A0A133NL16 A0A133NL09 A0A133NG83 A0A133NG69 A0A133NGF1 A0A133NGM1 A0A133NGF7 A0A133NLV9 A0A133NI88 A0A133NIG9 A0A133NGI4 A0A133NHK1 A0A133NLI2

Detected only *in G. vaginalis*	3	A0A133NK82 A0A133NG90 A0A133NMB1
Detected only *in G. piotii*	120	A0A133NMA4 A0A133NHZ3 A0A133NFX5 A0A133NHS1 A0A133NI63 A0A133NI39 A0A133NL34 A0A133NI49 A0A133NI29 A0A133NHY6 A0A133NLU3 A0A133NFS5 A0A133NH57 A0A133NJ83 A0A133NL04 A0A133NI07 A0A133NK44 A0A133NGJ9 A0A133NI01 A0A133NGD9 A0A133NID2 A0A133NG14 A0A133NMN7 A0A133NGL4 A0A133NGL3 A0A133NID3 A0A133NI03 A0A133NJ50 A0A133NHG2 A0A133NHE8 A0A133NLQ4 A0A133NKN4 A0A133NG37 A0A133NL64 A0A133NHW0 A0A133NFT8 A0A133NJ11 A0A133NL11 A0A133NFX1 A0A133NIE3 A0A133NKN3 A0A133NH99 A0A133NMF1 A0A133NFT4 A0A133NHQ8 A0A133NJ19 A0A133NFY5 A0A133NI09 A0A133NL83 A0A133NIW8 A0A133NHZ5 A0A133NLJ2 A0A133NMT8 A0A133NL12 A0A133NL80 A0A133NGF3 A0A133NI21 A0A133NFN4 A0A133NIZ6 A0A133NJE5 A0A133NK70 A0A133NG56 A0A133NKS0 A0A133NI19 A0A133NL25 A0A133NGM5 A0A133NG04 A0A133NFV2 A0A133NIC5 A0A133NK98 A0A133NIX3 A0A133NFX3 A0A133NI73 A0A133NKJ8 A0A133NG98 A0A133NI28 A0A133NH89 A0A133NIQ7 A0A133NGI9 A0A133NI54 A0A133NG13 A0A133NHG6 A0A133NGN6 A0A133NFQ7 A0A133NL48 A0A133NHS4 A0A133NH38 A0A133NIE8 A0A133NI12 A0A133NJ86 A0A133NJA7 A0A133NJF2 A0A133NL97 A0A133NGA4 A0A133NLP8 A0A133NHF8 A0A133NL17 A0A133NI16 A0A133NLG9 A0A133NI17 A0A133NI37 A0A133NI11 A0A133NLH9 A0A133NL75 A0A133NL54 A0A133NKL7 A0A133NML2 A0A133NG30 A0A133NK32 A0A133NL36 A0A133NFY0 A0A133NLC0 A0A133NFS0 A0A133NJ38 A0A133NFZ1 A0A133NL39 A0A133NFR9 A0A133NK38 A0A133NGW4 A0A133NKM4
Detected only *in G. leopoldii*	4	A0A133NLH3 A0A133NLF6 A0A133NG46 A0A133NL40

Table S3. Description of proteins associated with bacterial virulence identified across the proteomes of the studied *G. leopoldii* and/or *G. piotii* and/or *G. vaginalis* biofilms.

Accession	Gene	Description
**Cytotoxicity**		
E3D7C1	HMPREF0421_20066	Thiol-activated cytolysin OS=Gardnerella vaginalis (strain ATCC 14019 / 317) OX=525284 GN=HMPREF0421_20066 PE=3 SV=1
D2RCU3	HMPREF0424_0103	Thiol-activated cytolysin OS=Gardnerella vaginalis (strain 409-05) OX=553190 GN=HMPREF0424_0103 PE=3 SV=1
**Biofilm Formation**		
E3D8T2	HMPREF0421_20394	peptidoglycan glycosyltransferase OS=Gardnerella vaginalis (strain ATCC 14019 / 317) OX=525284 GN=HMPREF0421_20394 PE=4 SV=1
S4HBR8	HMPREF1580_00255	Glycosyltransferase group 1 family protein OS=Gardnerella vaginalis JCP8070 OX=1261065 GN=HMPREF1580_00255 PE=4 SV=1
S4HQL1	HMPREF1580_00784	peptidoglycan glycosyltransferase OS=Gardnerella vaginalis JCP8070 OX=1261065 GN=HMPREF1580_00784 PE=4 SV=1
S4HCH0	HMPREF1580_00102	Glycosyltransferase group 2 family protein OS=Gardnerella vaginalis JCP8070 OX=1261065 GN=HMPREF1580_00102 PE=4 SV=1
S4HNM6	HMPREF1580_01090	peptidoglycan glycosyltransferase OS=Gardnerella vaginalis JCP8070 OX=1261065 GN=HMPREF1580_01090 PE=4 SV=1

D6SW61	GVAMD_0934	peptidoglycan glycosyltransferase OS=Gardnerella vaginalis AMD OX=682147 GN=GVAMD_0934 PE=4 SV=1
D6SYT9	GVAMD_1136	Glycosyltransferase OS=Gardnerella vaginalis AMD OX=682147 GN=GVAMD_1136 PE=4 SV=1
D6SVC3	GVAMD_0033	peptidoglycan glycosyltransferase OS=Gardnerella vaginalis AMD OX=682147 GN=GVAMD_0033 PE=4 SV=1
D6SYU2	GVAMD_1133	Glycosyltransferase 2-like domain-containing protein OS=Gardnerella vaginalis AMD OX=682147 GN=GVAMD_1133 PE=4 SV=1
D6SYU3	GVAMD_1132	Cell wall biogenesis glycosyltransferase OS=Gardnerella vaginalis AMD OX=682147 GN=GVAMD_1132 PE=4 SV=1
D6SXS1	GVAMD_0396	Glycosyltransferase OS=Gardnerella vaginalis AMD OX=682147 GN=GVAMD_0396 PE=4 SV=1
S4HB82	HMPREF1580_00333	Glycosyltransferase group 2 family protein OS=Gardnerella vaginalis JCP8070 OX=1261065 GN=HMPREF1580_00333 PE=4 SV=1
D2RC02	HMPREF0424_1138	Glycosyltransferase group 2 family protein OS=Gardnerella vaginalis (strain 409-05) OX=553190 GN=HMPREF0424_1138 PE=4 SV=1

E3D954	HMPREF0421_20516	Cell wall synthesis protein Wag31 OS=Gardnerella vaginalis (strain ATCC 14019 / 317) OX=525284 GN=HMPREF0421_20516 PE=3 SV=1
S4HPA2	HMPREF1580_00212	Cell wall synthesis protein Wag31 OS=Gardnerella vaginalis JCP8070 OX=1261065 GN=HMPREF1580_00212 PE=3 SV=1
D2RBY3	HMPREF0424_1117	Cell wall synthesis protein Wag31 OS=Gardnerella vaginalis (strain 409-05) OX=553190 GN=HMPREF0424_1117 PE=3 SV=1
D6SYJ8	GVAMD_0525	Gram-positive cocci surface proteins LPxTG domain-containing protein OS=Gardnerella vaginalis AMD OX=682147 GN=GVAMD_0525 PE=4 SV=1
S4IXE1	HMPREF1580_00620	LPXTG-motif protein cell wall anchor domain protein OS=Gardnerella vaginalis JCP8070 OX=1261065 GN=HMPREF1580_00620 PE=4 SV=1
S4HN27	HMPREF1580_01154	LPXTG-motif protein cell wall anchor domain protein OS=Gardnerella vaginalis JCP8070 OX=1261065 GN=HMPREF1580_01154 PE=4 SV=1
S4H5G7	HMPREF1580_01250	LPXTG-motif protein cell wall anchor domain protein OS=Gardnerella vaginalis JCP8070 OX=1261065 GN=HMPREF1580_01250 PE=4 SV=1

S4H9V4	HMPREF1580_00596	LPXTG-motif protein cell wall anchor domain protein OS=Gardnerella vaginalis JCP8070 OX=1261065 GN=HMPREF1580_00596 PE=4 SV=1
S4HEQ0	HMPREF1580_01153	LPXTG-motif protein cell wall anchor domain protein OS=Gardnerella vaginalis JCP8070 OX=1261065 GN=HMPREF1580_01153 PE=4 SV=1
S4HKB9	HMPREF1580_00871	LPXTG-motif protein cell wall anchor domain protein OS=Gardnerella vaginalis JCP8070 OX=1261065 GN=HMPREF1580_00871 PE=4 SV=1
E3D938	HMPREF0421_20500	LPXTG-motif cell wall anchor domain protein OS=Gardnerella vaginalis (strain ATCC 14019 / 317) OX=525284 GN=HMPREF0421_20500 PE=4 SV=1
D6SWZ2	GVAMD_1211	Sortase (Surface protein transpeptidase) OS=Gardnerella vaginalis AMD OX=682147 GN=GVAMD_1211 PE=4 SV=1
S4HK20	HMPREF1580_00299	Sortase family protein OS=Gardnerella vaginalis JCP8070 OX=1261065 GN=HMPREF1580_00299 PE=4 SV=1
D2RCM3	HMPREF0424_0029	Sortase family protein OS=Gardnerella vaginalis (strain 409-05) OX=553190 GN=HMPREF0424_0029 PE=4 SV=1
**Glycogen cleaving**		

D6SV67	GVAMD_0052	Type II secretory pathway pullulanase PulA glycosidase OS=Gardnerella vaginalis AMD OX=682147 GN=GVAMD_0052 PE=4 SV=1
D6SVD5	GVAMD_0045	Alpha-glucosidase (Family 31 of glycosyl hydrolases) OS=Gardnerella vaginalis AMD OX=682147 GN=GVAMD_0045 PE=3 SV=1
S4HH81	HMPREF1580_00766	Pullulanase type I OS=Gardnerella vaginalis JCP8070 OX=1261065 GN=HMPREF1580_00766 PE=4 SV=1
S4ITB8	HMPREF1580_01211	Glycogen debranching enzyme GlgX OS=Gardnerella vaginalis JCP8070 OX=1261065 GN=HMPREF1580_01211 PE=3 SV=1
S4HNK6	HMPREF1580_00346	Alpha-amylase OS=Gardnerella vaginalis JCP8070 OX=1261065 GN=HMPREF1580_00346 PE=3 SV=1
E3D7L7	pulA	Alpha-amylase OS=Gardnerella vaginalis (strain ATCC 14019 / 317) OX=525284 GN=pulA PE=3 SV=1
S4HE71	HMPREF1580_01197	Alpha-amylase OS=Gardnerella vaginalis JCP8070 OX=1261065 GN=HMPREF1580_01197 PE=3 SV=1
S4H990	HMPREF1580_00663	Alpha amylase catalytic domain protein OS=Gardnerella vaginalis JCP8070 OX=1261065 GN=HMPREF1580_00663 PE=4 SV=1

E3D892	HMPREF0421_20243	Alpha amylase catalytic domain protein OS=Gardnerella vaginalis (strain ATCC 14019 / 317) OX=525284 GN=HMPREF0421_20243 PE=4 SV=1
D6SYA1	GVAMD_0260	Alpha-amylase OS=Gardnerella vaginalis AMD OX=682147 GN=GVAMD_0260 PE=3 SV=1
E3D855	HMPREF0421_20204	Alpha amylase catalytic domain protein OS=Gardnerella vaginalis (strain ATCC 14019 / 317) OX=525284 GN=HMPREF0421_20204 PE=4 SV=1
**Immune Evasion**		
D2RD34	HMPREF0424_0197	Macrophage migration inhibitory factor OS=Gardnerella vaginalis (strain 409-05) OX=553190 GN=HMPREF0424_0197 PE=4 SV=1
S4HKW2	HMPREF1580_00064	Alkyl hydroperoxide reductase C OS=Gardnerella vaginalis JCP8070 OX=1261065 GN=HMPREF1580_00064 PE=3 SV=1
D2RCK0	ahpC	Alkyl hydroperoxide reductase C OS=Gardnerella vaginalis (strain 409-05) OX=553190 GN=ahpC PE=3 SV=1
**Iron Acquisition**		
S4H9V8	HMPREF1580_00601	Iron permease FTR1 family protein OS=Gardnerella vaginalis JCP8070 OX=1261065 GN=HMPREF1580_00601 PE=3 SV=1

D2RCK7	HMPREF0424_0013	Ferritin-like protein OS=Gardnerella vaginalis (strain 409-05) OX=553190 GN=HMPREF0424_0013 PE=3 SV=1
S4HHJ3	HMPREF1580_00689	Ferritin-like protein OS=Gardnerella vaginalis JCP8070 OX=1261065 GN=HMPREF1580_00689 PE=3 SV=1
D2RCA0	HMPREF0424_1242	Isochorismatase family protein OS=Gardnerella vaginalis (strain 409-05) OX=553190 GN=HMPREF0424_1242 PE=4 SV=1
D6SWK3	GVAMD_1076	High-affinity Fe2+/Pb2+ permease OS=Gardnerella vaginalis AMD OX=682147 GN=GVAMD_1076 PE=3 SV=1
D6SWK2	GVAMD_1075	High-affinity Fe2+ transport protein OS=Gardnerella vaginalis AMD OX=682147 GN=GVAMD_1075 PE=3 SV=1
S4H775	HMPREF1580_00961	FeS assembly protein SufD OS=Gardnerella vaginalis JCP8070 OX=1261065 GN=HMPREF1580_00961 PE=4 SV=1
**Quorum sensing**		
E3DAA1	luxS	S-ribosylhomocysteine lyase OS=Gardnerella vaginalis (strain ATCC 14019 / 317) OX=525284 GN=luxS PE=3 SV=1
**Adhesion**		
S4HJT4	HMPREF1580_00916	Adhesin domain-containing protein OS=Gardnerella vaginalis JCP8070 OX=1261065 GN=HMPREF1580_00916 PE=4 SV=1

D6SXP4	GVAMD_0369	ABC-type metal ion transporter periplasmic component/surface adhesin OS=Gardnerella vaginalis AMD OX=682147 GN=GVAMD_0369 PE=4 SV=1
D6SYB3	GVAMD_0272	ABC-type metal ion transporter periplasmic component/surface adhesin OS=Gardnerella vaginalis AMD OX=682147 GN=GVAMD_0272 PE=4 SV=1
D6SX96	GVAMD_0097	Type-1 fimbrial major subunit OS=Gardnerella vaginalis AMD OX=682147 GN=GVAMD_0097 PE=4 SV=1
**Antibiotic Resistance**		
D6SV95	GVAMD_0005	ABC-type multidrug transporter ATPase and permease OS=Gardnerella vaginalis AMD OX=682147 GN=GVAMD_0005 PE=4 SV=1
D6SYP5	GVAMD_0478	ABC-type multidrug transporter ATPase component OS=Gardnerella vaginalis AMD OX=682147 GN=GVAMD_0478 PE=4 SV=1
D6SWS2	GVAMD_1186	ABC-type multidrug transporter ATPase component OS=Gardnerella vaginalis AMD OX=682147 GN=GVAMD_1186 PE=4 SV=1
D6SWR9	GVAMD_1189	ABC-type multidrug transporter ATPase component OS=Gardnerella vaginalis AMD OX=682147 GN=GVAMD_1189 PE=4 SV=1
D6SYF1	GVAMD_0310	ABC-type multidrug transporter ATPase component OS=Gardnerella vaginalis AMD OX=682147 GN=GVAMD_0310 PE=3 SV=1

D6SYM8	GVAMD_0495	ABC-type bacteriocin/lantibiotic exporter OS=Gardnerella vaginalis AMD OX=682147 GN=GVAMD_0495 PE=4 SV=1
D6SYF5	GVAMD_0314	ABC-type bacteriocin/lantibiotic exporter OS=Gardnerella vaginalis AMD OX=682147 GN=GVAMD_0314 PE=4 SV=1
S4HKG1	HMPREF1580_00192	Aminoglycoside phosphotransferase domain-containing protein OS=Gardnerella vaginalis JCP8070 OX=1261065 GN=HMPREF1580_00192 PE=4 SV=1
D2RA54	HMPREF0424_0444	Aminoglycoside phosphotransferase domain-containing protein OS=Gardnerella vaginalis (strain 409-05) OX=553190 GN=HMPREF0424_0444 PE=4 SV=1

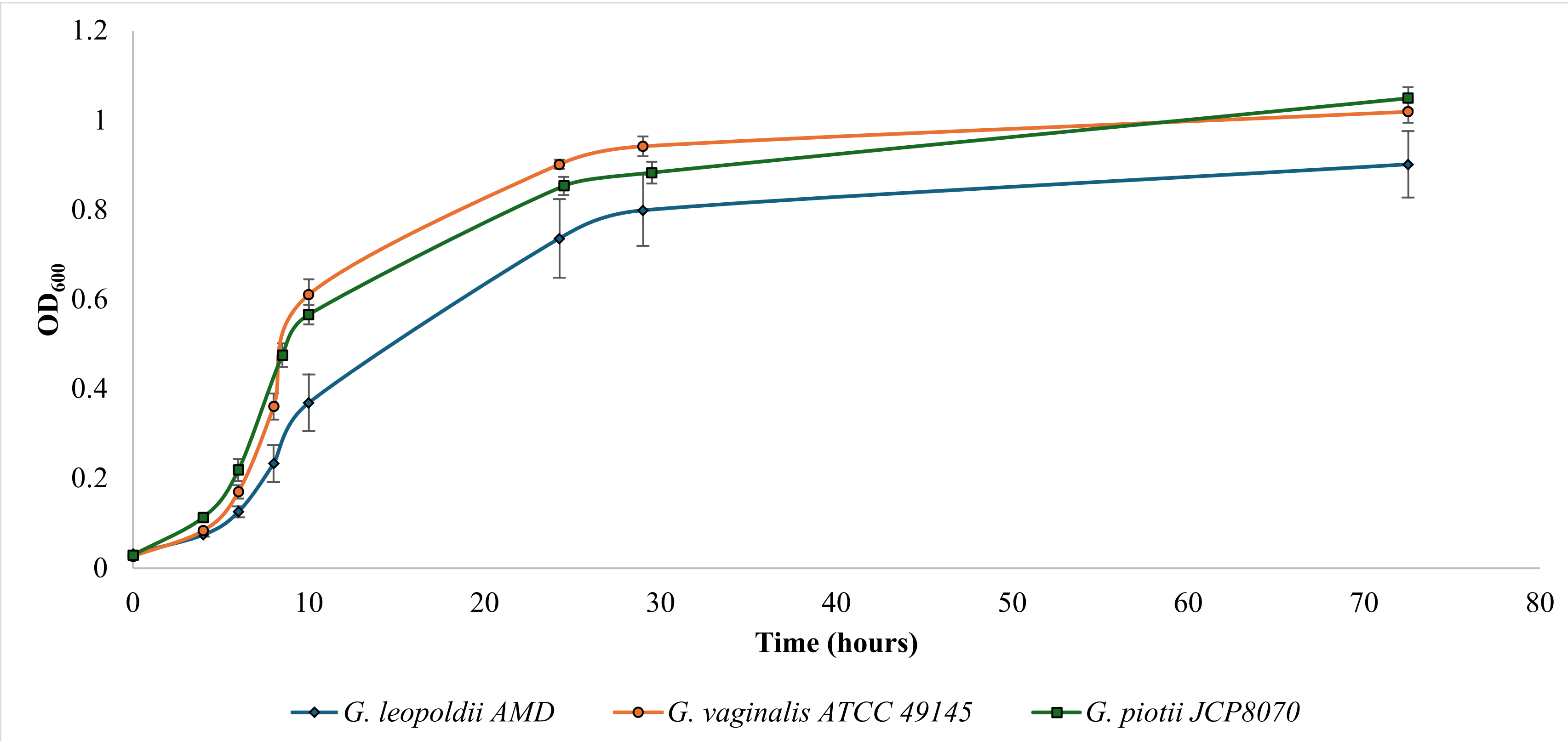

Fig. S1. Growth curves of *Gardnerella* species isolates grown in NYC III broth over a 72-hour period. Marked OD_{600} values indicate the means across all biological and technical replicates, with the error bars representing the standard deviation

Table S4. ***cpn*60 primers used for sequencing of *Gardnerella* isolates.**

Name	Primer Sequence (5' to 3')	References
**H729**	CGCCAGGGTTTTCCCAGTCACGACGAIIIIGCIGGIGAYGGIACIACIAC	(1)
**H1134**	TAATACGACTCACTATAGGGYKIYKITCICCRAAICCIGGIGCY	(2)

References:

1. Paramel Jayaprakash T, Schellenberg JJ, Hill JE. Resolution and characterization of distinct cpn60-based subgroups of Gardnerella vaginalis in the vaginal microbiota. PLoS One. 2012;7(8):e43009.
2. Lukwinski AT, Hill JE, Khachatourians GG, Hemmingsen SM, Hegedus DD. Biochemical and taxonomic characterization of bacteria associated with the crucifer root maggot (Delia radicum). Can J Microbiol. 2006;52(3):197-208.
